# Polarized epidermal growth factor secretion ensures robust vulval cell fate specification in *Caenorhabditis elegans*

**DOI:** 10.1242/dev.175760

**Published:** 2020-06-04

**Authors:** Louisa Mereu, Matthias K. Morf, Silvan Spiri, Peter Gutierrez, Juan M. Escobar-Restrepo, Michael Daube, Michael Walser, Alex Hajnal

**Affiliations:** 1 Department of Molecular Life Sciences, University of Zürich, Winterthurerstrasse 190, CH-8057 Zürich, Switzerland; 2 Molecular Life Science PhD Program, University and ETH Zürich, CH-8057 Zürich, Switzerland

**Keywords:** *C. elegans*, Epidermal growth factor, Polarity, Cell fate

## Abstract

The anchor cell (AC) in *C. elegans* secretes an epidermal growth factor (EGF) homolog that induces adjacent vulval precursor cells (VPCs) to differentiate. The EGF receptor in the nearest VPC sequesters the limiting EGF amounts released by the AC to prevent EGF from spreading to distal VPCs. Here, we show that not only EGFR localization in the VPCs but also EGF polarity in the AC is necessary for robust fate specification. The AC secretes EGF in a directional manner towards the nearest VPC. Loss of AC polarity causes signal spreading and, when combined with MAPK pathway hyperactivation, the ectopic induction of distal VPCs. In a screen for genes preventing distal VPC induction, we identified *sra-9* and *nlp-26* as genes specifically required for polarized EGF secretion. *sra-9(lf)* and *nlp-26(lf)* mutants exhibit errors in vulval fate specification, reduced precision in VPC to AC alignment and increased variability in MAPK activation. *sra-9* encodes a seven-pass transmembrane receptor acting in the AC and *nlp-26* a neuropeptide-like protein expressed in the VPCs. SRA-9 and NLP-26 may transduce a feedback signal to channel EGF secretion towards the nearest VPC.

## Abstract (precis)

**Summary:** The *sra-9* and *nlp-26* genes are required for the polarized and directional secretion of the epidermal growth factor during vulval development in *C. elegans*.

## INTRODUCTION

Intercellular communication relies on the spatially and temporally controlled release of signaling molecules by signal-emitting cells. Members of the epidermal growth factor (EGF) family are involved in a variety of cell fate decisions in all metazoans (Massagué and Pandiella, 1993). Secreted EGF ligands bind to receptor tyrosine kinases of the ErbB/EGFR family, which activate different intracellular signaling pathways, such as the RAS/MAPK, PI3K/AKT, JAK/STAT and the PLC pathways, depending on the cellular context (Hynes and MacDonald, 2009). Although many studies have focused on the mechanisms controlling the polarized secretion, internalization and recycling of EGF receptors (Sorkin and Goh, 2008), less is known about the factors controlling the intracellular trafficking of the EGF family ligands. EGF ligands are typically produced as transmembrane precursor proteins. They can either act as a membrane-bound form in a juxtacrine manner or be cleaved by intracellular rhomboid family proteases (Urban et al., 2001) and extracellular metalloproteases, allowing them to be released and signal at a distance (Massagué and Pandiella, 1993). The concentration and temporal duration of an EGF signal can lead to differential responses in the signal-receiving cells, for example during cell fate specification (Katz et al., 1995), apoptosis (Iwamoto et al., 1999) or cell migration (Wyckoff et al., 2004). Moreover, by controlling the subcellular localization of EGF ligands, cells can regulate ligand availability and add directionality to the signal (Dempsey et al., 1997). The basolateral versus apical sorting of transforming growth factor alpha (TGFα) in cultured epithelial cells is important for restricting EGFR activation to the basolateral compartment (Singh and Coffey, 2014). One of the few examples demonstrating polarized EGF secretion during animal development *in vivo* is the release of the *Drosophila* EGF ligand Spitz by photoreceptor neurons (Yogev et al., 2010).

**Fig. 1.**
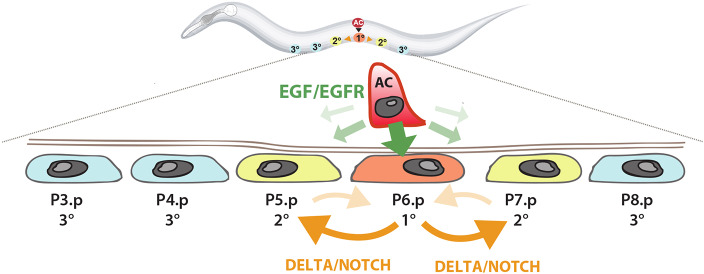
**Model of vulval development.** The AC releases LIN-3 to induce the 1° cell fate in the most proximal VPC P6.p. The lateral DELTA signal from P6.p specifies the 2° fate in the adjacent VPCs P5.p and P7.p, whereas the distal VPCs P3.p, P4.p and P8.p adopt the uninduced 3° fate.

The *Caenorhabditis elegans* genome encodes a single EGF-like growth factor, termed LIN-3, and an EGFR receptor homolog, termed LET-23 (Sundaram, 2013). Thus, the lack of genetic redundancy has greatly simplified analysis of the EGF signaling network in *C. elegans*. The LIN-3/LET-23 pathway controls many cell fate decisions during larval development as well as various adult functions. In particular, development of the *C. elegans* hermaphrodite vulva is an excellent model for studying cell fate decisions controlled by EGF signaling at single cell resolution (Sternberg, 2005). During the first larval stage (L1), 12 epidermal Pn.p cells (P1.p to P12.p) are generated in the ventral epidermis (Fig. 1). Six Pn.p cells in the mid-body region (P3.p to P8.p) are specified by a Wingless signal to become vulval precursor cells (VPCs) that express LET-23 and are competent to differentiate into vulval cells (Eisenmann et al., 1998). VPC differentiation is induced by the uterine anchor cell (AC) located dorsally to the VPCs in the somatic gonad (Kimble, 1981). From the L2 stage on, the AC expresses LIN-3 (Hill and Sternberg, 1992). Even though LIN-3 is secreted to the plasma membrane as a type I transmembrane protein similar to TGFα, the AC can induce distant VPCs without making direct contact (Hill and Sternberg, 1992). Thus, at least a fraction of LIN-3 must be released from the AC, which probably occurs by shedding of the extracellular domain rather than by intracellular proteolysis, as the intracellular rhomboid protease ROM-1 does not act in the AC (Dutt et al., 2004). The LIN-3 signal activates, via its receptor LET-23, the RAS/MAPK pathway, which specifies the primary (1°) cell fate in the nearest VPC P6.p (Fig. 1) (Sternberg, 2005; Sundaram, 2013). Strong RAS/MAPK signaling in P6.p leads to the upregulation of LET-23 and of DSL family NOTCH ligands (Chen and Greenwald, 2004; Greenwald and Kovall, 2013). The latter activate the LIN-12 NOTCH receptor in the adjacent VPCs (P5.p and P7.p) by lateral signaling to repress the 1° and induce the secondary (2°) cell fate (Berset et al., 2001; Yoo et al., 2004). The distal VPCs (P3.p, P4.p and P8.p) that receive neither the inductive LIN-3 nor the lateral DSL signal adopt the uninduced, tertiary (3°) cell fate. The 3° VPCs divide once and then fuse with the surrounding epidermis (hypodermal syncytium; hyp7). This interplay between the EGFR/RAS/MAPK and NOTCH pathways results in an invariant 2°-1°-2° vulval cell fate pattern.

Vulval induction in wild-type larvae is extremely robust (Félix and Barkoulas, 2012). P6.p, the VPC located closest to the AC adopts the 1° cell fate when animals are raised under standard conditions. Rare errors in vulval fate specification, such as shifts in the 1° fate from P6.p to another VPC or the hyperinduction of extra VPCs, only occur when animals are grown under suboptimal conditions, for example at low temperature or under food starvation (Braendle and Félix, 2008). The AC produces a limiting amount of the inductive LIN-3 signal, whereas LET-23 expressed by the VPCs provides excess LIN-3 binding sites (Barkoulas et al., 2013; Simske and Kim, 1995). Hence, the nearest VPC P6.p sequesters most of the inductive signal and thereby prevents induction of additional, more distal VPCs (Hajnal et al., 1997). Efficient LIN-3 sequestering and vulval induction both require the localization of LET-23 to the basolateral membrane compartment of the VPCs facing the AC (Whitfield et al., 1999). Basolateral LET-23 localization is maintained by the tripartite LIN-2/LIN-7/LIN-10 receptor localization complex (Kaech et al., 1998). The apical mislocalization of LET-23, for example in *lin-2(0)* mutants, leads to a strong reduction in vulval induction and a penetrant vulvaless (Vul) phenotype (Hoskins et al., 1996). However, in combination with a loss-of-function (*lf*) mutation in the RAS GTPase-activating protein GAP-1, which causes mild hyperactivation of the RAS/MAPK pathway, apical LET-23 mislocalization causes ectopic induction of VPCs distant from the AC and a multivulva (Muv) phenotype (Hajnal et al., 1997). This finding has been attributed to an expanded range of the LIN-3 signal caused by loss of LIN-3 sequestering by P6.p.

Here, we show that not only basolateral LET-23 localization in the VPCs, but also the polarized secretion of LIN-3 by the AC towards the 1° VPC, is necessary for robust vulval induction. Furthermore, an unbiased screen for genes controlling polarized LIN-3 secretion identified a putative neuropeptide-like ligand and a GPCR required for LIN-3 polarity and robust vulval induction.

## RESULTS

### Depolarization of the AC causes ectopic and shifted vulval induction

**Fig. 2.**
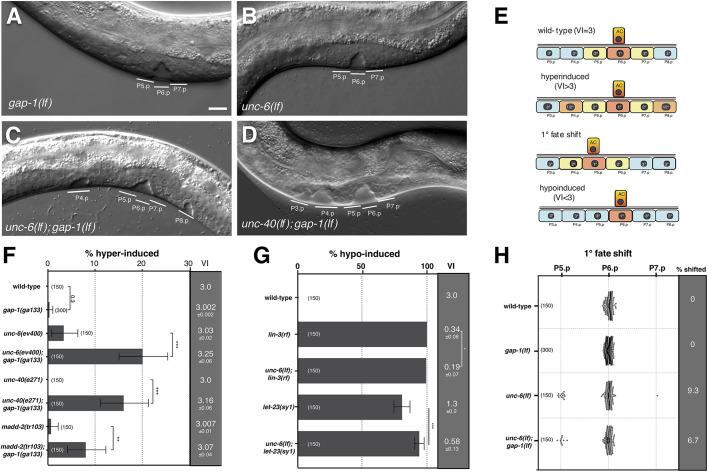
**AC polarity is necessary for robust vulval induction.** (A-D) Nomarski images of mid-L4 stage larvae of the indicated genotypes. The white lines indicate the descendants of induced VPCs that formed an invagination. (E) Scheme of the different observed vulval phenotypes: wild-type (VI=3), hyperinduced (VI>3), 1° fate shift, and hypo-induced (VI<3). (F,G) Percentage of animals of the indicated genotypes with a hyperinduced (F) or hypo-induced (G) vulval phenotype. The gray columns to the right show the VI value (mean±s.e.m.). Error bars indicate the 95% confidence intervals calculated by bootstrapping with a resampling size of 10,000. Statistical significance was calculated using a *t*-test for independent samples. (H) Animals with a 1° cell fate shift from P6.p to P5.p or P7.p. For each genotype, the percentage of animals showing a 1° fate shift is shown in the gray column to the right. The numbers in brackets in each plot refer to the numbers of animals scored. *P<0.05, **P<0.01, ***P<0.001. Scale bar: 10 µm.

In wild-type *C. elegans* larvae, the three proximal VPCs (P5.p, P6.p and P7.p) are induced to adopt vulval cell fates. Thus, the vulval induction index (VI) in the wild-type is exactly 3. An *lf* mutation in the RAS-GAP gene *gap-1(ga133lf)* enhances the activity of the inductive RAS/MAPK signaling pathway, but not enough to cause induction of additional VPCs (Hajnal et al., 1997). Only one in 300 *gap-1(lf)* animals exhibited a hyperinduced phenotype (Fig. 2A,E,F). *gap-1(lf)* can thus be utilized as a sensitized genetic background to detect changes in LIN-3 distribution, such as the reduced LIN-3 sequestering caused by apical LET-23 mislocalization (Hajnal et al., 1997).

The *unc-6* gene encodes a Netrin homologue that is secreted by ventral cord motor neurons and polarizes the AC along the dorso-ventral axis towards the VPCs that are aligned on the ventral midline (Ziel et al., 2009). Surprisingly, *unc-6(ev400lf); gap-1(ga133lf)* double mutants exhibited a 20% penetrant hyperinduced phenotype as a result of ectopic induction of distal VPCs (Fig. 2C,F). Similar hyperinduced phenotypes were observed after combining *gap-1(lf)* with mutations in other known AC polarity regulators, such as the Netrin receptor *unc-40* (Ziel et al., 2009) or the *unc-40* downstream effector *madd-2* (Fig. 2D,F) (Morf et al., 2013). Furthermore, depolarization of the AC by *unc-6(lf)* enhanced the Vul phenotype and reduced the VI of the *lin-3(e1417)* reduction-of-function allele or the *let-23(sy1)* receptor mislocalization mutant (Fig. 2E,G).

To test whether the hyperinduced vulval phenotype is caused by depolarization of the AC, we introduced an *unc-40::gfp* rescue minigene (*zhEx668*) driven by the AC-specific *mk62-63 cdh-3* promoter fragment (Ziel et al., 2009) into the *unc-40(lf); gap-1(lf)* background. The *unc-40::gfp* transgene was expressed in the AC and in ventral nerve cord (VNC) neurons of mid-L2 to early L3 larvae, but not in the VPCs (Fig. S1A,B). Three independent transgenic lines exhibited partial suppression of the hyperinduced phenotype and a reduced VI for *unc-40(lf); gap-1(lf)* double mutants (Fig. S1C). Because VNCs neurons are unlikely to affect VPC differentiation, *unc-40* probably acts in the AC to inhibit vulval induction.

In wild-type larvae, the AC invariably induces the most proximal VPC (P6.p) to adopt the 1° cell fate (Braendle and Félix, 2008). Besides the changes in vulval induction, we also observed shifts of the 1° fate from P6.p to P5.p, or rarely to P7.p, in *unc-6(lf)* single mutants as well as in *unc-6(lf); gap-1(lf)* double mutants (Fig. 2E,H).

These findings suggested that dorso-ventral AC polarity is needed for the robust induction of the 1° fate in P6.p. Similar to the case of apical LET-23 mislocalization, loss of AC polarity may affect the range and distribution of the inductive LIN-3 signal among the VPCs.

### Polarized distribution of LIN-3 in the AC

**Fig. 3.**
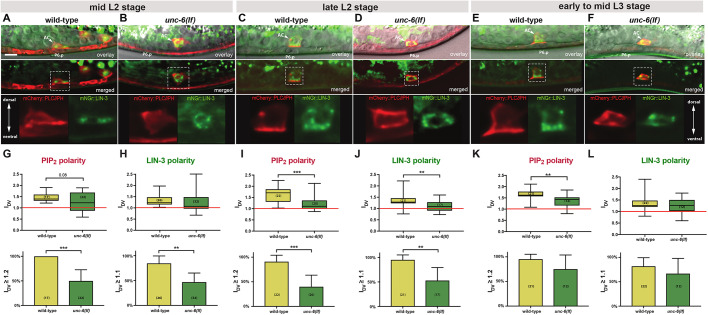
**LIN-3 expression in the AC is polarized and requires the UNC-6 Netrin signal.** (A,B) Top row: Nomarski images of a mid-sagittal plane overlaid with summed *z*-projections of the *P_cdh3_::mCherry::PLCδ^PH^* (red) and the *mNGr::lin-3* (green) reporters in wild-type (A) and *unc-6(lf)* (B) mid-L2 larvae. Positions of the AC (arrowheads) and the P6.p nuclei are indicated. The middle row shows the merged *z*-projections of the *P_cdh3_::mCherry::PLCδ^PH^* (red) and the *mNGr::lin-3* (green) reporters alone. The dashed boxes outline the AC regions magnified about 2.5-fold in the bottom row. (C-F) Reporter expression in wild-type and *unc-6(lf)* late L2 larvae (C,D) and early to mid-L3 larvae (E,F). Animals were staged according to their gonad length as described in the Materials and Methods section. (G,H) Box plots of the PIP_2_ (mCherry::PLCδ^PH^) (G) and LIN-3 (H) polarity indices I_DV_ in wild-type and *unc-6(lf)* mid-L2 larvae. Error bars indicate the minimum and maximum values. Significance was calculated using a *t*-test for independent samples of unequal variance. The graphs underneath the box plots show the percentage of animals with a PIP_2_ I_DV_>1.2 and a LIN-3 I_DV_>1.1. These threshold values correspond approximately to the 10th percentile for the two markers. Error bars indicate the 95% confidence intervals. Statistical significance was calculated using the nonparametric, unpaired Mann–Whitney test. (I-L) The same analysis for late L2 (I,J) and early to mid-L3 (K,L) larvae. The numbers in brackets in each graph refer to the numbers of animals scored for each genotype and stage. **P<0.01, ***P<0.001. Scale bar: 5 µm.

One possible explanation for the vulval phenotypes observed in AC polarity mutants is that the AC normally secretes LIN-3 in a polarized fashion in the direction of the closest VPC. To directly observe LIN-3 localization in the AC, we inserted an *mNeongreen* (*mNGr*) fluorophore cassette into the *lin-3* locus directly after the predicted signal sequence (after Leu24 in LIN-3A) and N-terminally to the EGF domain, using CRISPR/Cas9-mediated genome engineering [*mNGr::lin-3(zh112)*, Fig. S2] (Dickinson et al., 2015). *mNGr::lin-3(zh112)* animals exhibited an overall wild-type phenotype without any obvious defects in vulval development, indicating that the mNGr::LIN-3 fusion protein retains its activity at levels comparable with the wild-type LIN-3 protein. In L2 and L3 larvae, mNGr::LIN-3 expression was detected in intracellular punctae and on the basal cortex of the AC (Fig. 3A,C,E). No extracellular mNGr::LIN-3 signal could be detected, suggesting that the majority of mNGr::LIN-3 remains attached to the AC surface after its secretion to the plasma membrane, or that a cleavage product of the mNGr::LIN-3 protein is released from the AC and rapidly taken up by the adjacent VPCs or degraded. The same localization pattern was observed with a multicopy GFP::LIN-3 transgene (*zhIs67*), in which the GFP tag had been inserted at the same position as in the endogenous *mNGr::lin-3(zh112)* reporter strain (Fig. S3).

In wild-type larvae, mNGr::LIN-3 was enriched towards the ventral cortex of the AC from the mid-L2 stage until the mid-L3 stage (Fig. 3A,C,E). To quantify LIN-3 polarity, we recorded optical sections through the AC of animals expressing mNGr::LIN-3 together with the *mCherry::PLCδ^PH^* reporter that labels phosphatidylinositol-(4,5)-bisphosphate (PIP_2_)-rich membranes and serves as a global AC polarity marker (Ziel et al., 2009). After measuring the average intensities in summed *z*-projections in the ventral and dorsal half of the AC, we calculated for each animal a dorso-ventral polarity index (I_DV_) for both mNGr::LIN-3 and PIP_2_ by dividing the signal intensity in the ventral by the intensity in the dorsal half of the AC (see Materials and Methods). We first measured the mNGr::LIN-3 and PIP_2_ I_DV_ in wild-type larvae between the early L2 and mid-L3 stages before the first round of VPC divisions. The developmental stage was assigned according to the animal's gonad length (Kimble and Hirsh, 1979). The average mNGr::LIN-3 I_DV_ was around 1.3 at all three stages, with only a few cases showing an I_DV_ below 1.0 (Fig. 3H,J,L). Around 90% of the animals across the three stages analyzed had an I_DV_ higher than 1.1 (Fig. 3H,J,L). The PIP_2_ global polarity marker showed a slightly stronger polarization of the AC, with an average I_DV_ above 1.5 and an I_DV_≥1.2 in around 90% of the animals at the three stages analyzed (Fig. 3G,I,K).

Taken together, the endogenous *mNGr::lin-3* and transgenic *gfp::lin-3* reporters revealed a polarized distribution of LIN-3 in the AC before and during vulval induction, but no extracellular signal was detectable.

### LIN-3 polarity is reduced in *unc-6(lf)* mutants

We next tested whether global depolarization of the AC affects LIN-3 polarity. The *unc-6(ev400)* loss-of-function allele causes a strong reduction in AC polarity that manifests in a more uniform PIP_2_ localization. Accordingly, the average PIP_2_ I_DV_ was decreased in *unc-6(lf)* mutants from the mid-L2 stage on, such that fewer than 50% of the mid- to late L2 larvae had a PIP_2_ I_DV_ greater than 1.2 (Fig. 3B,D,G,I). PIP_2_ polarity remained reduced in early to mid-L3 *unc-6(lf)* larvae, although the effect was less pronounced (Fig. 3F,K).

LIN-3 polarity was also decreased in *unc-6(lf)* mutants (Fig. 3B,D,F). In mid- to late L2 larvae, the time period when the AC signal selects the 1° VPC, the LIN-3 I_DV_ was below 1.1 in around half of the *unc-6(lf)* mutants analyzed (Fig. 3H,J). By the mid-L3 stage, the effect of *unc-6(lf)* on LIN-3 polarity was less pronounced (Fig. 3L). Moreover, we observed a strong correlation between LIN-3 and PIP_2_ polarity on a per animal basis. There was a significant increase in the fraction of animals showing both PIP_2_ and LIN-3 depolarization in *unc-6(lf)* animals compared with the wild-type (Fig. S4).

We conclude that the polarized distribution of LIN-3 in the AC depends on the global polarity established by the UNC-6 Netrin signal.

### Whole-genome RNAi screen for ectopic vulval induction identifies genes specifically controlling LIN-3 polarity

The observation that depolarization of the AC in the sensitized *gap-1(lf)* background causes ectopic vulval induction and a Muv phenotype opened the possibility to conduct a systematic screen for global AC polarity regulators and possibly also for specific regulators of LIN-3 polarity. To this aim, we performed a genome-wide RNA interference (RNAi) screen in an *rrf-3(pk1426); gap-1(ga133)* hypersensitive background and searched for animals exhibiting a Muv phenotype (Simmer et al., 2002). Through this approach, we identified 51 primary candidate genes that showed a reproducible Muv phenotype upon RNAi knockdown in the *rrf-3(pk1426);gap-1(ga133)* background (Table S1). Among the candidates identified were three genes previously known to act in the UNC-6 Netrin pathway that polarizes the AC: *unc-40*, *unc-73* and *madd-2* (highlighted in green in Table S1) (Alexander et al., 2010; Morf et al., 2013; Ziel et al., 2009). To search for genes specifically regulating polarized LIN-3 localization in the AC, we screened again in a strain carrying the PIP_2_ global AC polarity marker together with the multicopy GFP::LIN-3 transgene *zhIs67* to increase signal intensity. This approach allowed us to identify candidates that specifically change LIN-3 distribution without affecting global AC polarity. Three of the 51 primary candidates exhibited altered GFP::LIN-3 but unchanged PIP_2_ localization after RNAi knockdown. Those genes were *sra-9*, *srh-247* and *nlp-26* (Table S1, highlighted in orange). Genes *sra-9* and *srh-247* encode putative seven-pass transmembrane G-protein coupled receptors (GPCRs) (Troemel et al., 1995); *nlp-26* encodes a secreted neuropeptide-like protein (Nathoo et al., 2001). However, a role during vulval development has not been reported for any of these genes so far. Because RNAi of *srh-247* showed a weaker and more variable phenotype, we focused further analyses on the *sra-9* and *nlp-26* genes.

### LIN-3 polarity is reduced in *sra-9* and *nlp-26* mutants, whereas general AC polarity is not altered

To confirm the RNAi phenotypes, we generated *sra-9(lf)* and *nlp-26(lf)* mutations using the CRISPR/Cas9 system (Arribere et al., 2014; Dickinson et al., 2015). The *sra-9(zh108)* allele contains a 2.5 kb deletion spanning the entire coding region. The *gfp::nlp-26(zh113)* allele carries a *gfp* insertion in the predicted signal peptide, generating a premature stop codon before the neuropeptide sequence. The endogenous *gfp:*:*nlp-26(zh113)* reporter did not show any GFP expression, indicating that NLP-26 is expressed at very low levels.

**Fig. 4.**
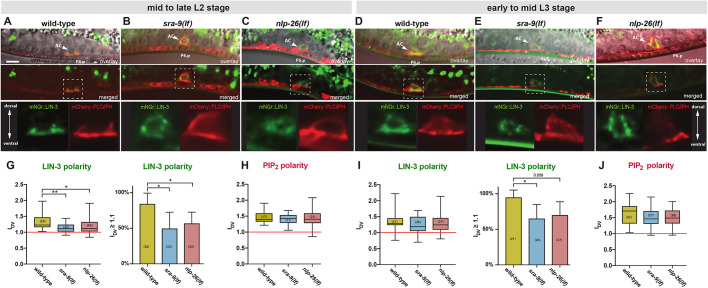
**SRA-9 and NLP-26 are required for LIN-3 polarity but not for global AC polarity.** (A-C) Top row: Nomarski images of a mid-sagittal plane overlaid with summed *z*-projections of the *P_cdh3_::mCherry::PLCδ^PH^* (red) and the *mNGr::lin-3* (green) reporters in wild-type (A), *sra-9(lf)* (B) and *nlp-26(lf)* (C) mid- to late-L2 larvae. The positions of the AC (arrowheads) and the P6.p nuclei are indicated. The middle row shows the merged *z*-projections of the *P_cdh3_::mCherry::PLCδ^PH^* (red) and the *mNGr::lin-3* (green) reporters alone. The dashed boxes outline the AC regions magnified about 2.5-fold in the bottom row. (D-F) Reporter expression in wild-type (D), *sra-9(lf)* (E) and *nlp-26(lf)* (F) early to mid-L3 larvae. Animals were staged according to their gonad length. (G-J) Box plots of the LIN-3 and PIP_2_ (mCherry::PLCδ^PH^) polarity indices I_DV_ in wild-type, *sra-9(lf)* and *nlp-26(lf)* larvae at the mid- to late L2 stage (G,H) and at the early to mid-L3 stage (I,J). Error bars indicate the minimum and maximum values. Statistical significance was calculated with a *t*-test for independent samples of unequal variance. The bar graphs beside the box plots in G and I show the percentage of animals with a LIN-3 I_DV_>1.1. Error bars indicate the 95% confidence intervals. Statistical significance was calculated using the nonparametric, unpaired Mann–Whitney test. The numbers in brackets in each graph refer to the numbers of animals scored for each genotype and stage. *P<0.05, **P<0.01. Scale bar: 5 µm.

We detected no significant differences in PIP_2_ polarity in *sra-9(lf)* or *nlp-26(lf)* mutants at the different stages analyzed (Fig. 4A-F,H,J). Thus, *sra-9* and *nlp-26* are not required for global AC polarity. However, LIN-3 polarity in *sra-9(lf)* and *nlp-26(lf)* mutants was significantly reduced at the mid- and late L2 stages, and to a lesser extent in early to mid-L3 larvae (Fig. 4A-F,G,I). In particular, the fraction of animals showing a LIN-3 I_DV_>1.1 was reduced in *sra-9(lf)* and *nlp-26(lf)* mutants (Fig. 4G,I)*.* After the mid-L3 stage, when the VPC fates had been determined and the first round of VPC division had been completed, there was no significant difference in LIN-3 polarity between *sra-9(lf)* and *nlp-26(lf)* mutants and the wild-type (data not shown). In contrast to *unc-6(lf)* mutants, LIN-3 and PIP_2_ polarity did not correlate in *sra-9(lf)* and *nlp-26(lf)* mutants at the level of individual animals. There was a significant increase in the fraction of animals with depolarized LIN-3 that maintained polarized PIP_2_ expression (Fig. S4). Thus, SRA-9 and NLP-26 are required for LIN-3 polarity independently of global AC polarity. Moreover, *sra-9(lf)* and *nlp-26(lf)* mutants did not show defects in AC invasion or vulval morphogenesis, two processes that depend on global AC polarity and are perturbed in *unc-6(lf)* mutants (Estes and Hanna-Rose, 2009; Ziel et al., 2009).

These results indicate that the asymmetric distribution of LIN-3 in the overall polarized AC is not the default state, but rather an actively regulated process.

### *sra-9* and *nlp-26* mutants exhibit an increased error rate in vulval induction and more variable MPK-1 ERK activity

**Fig. 5.**
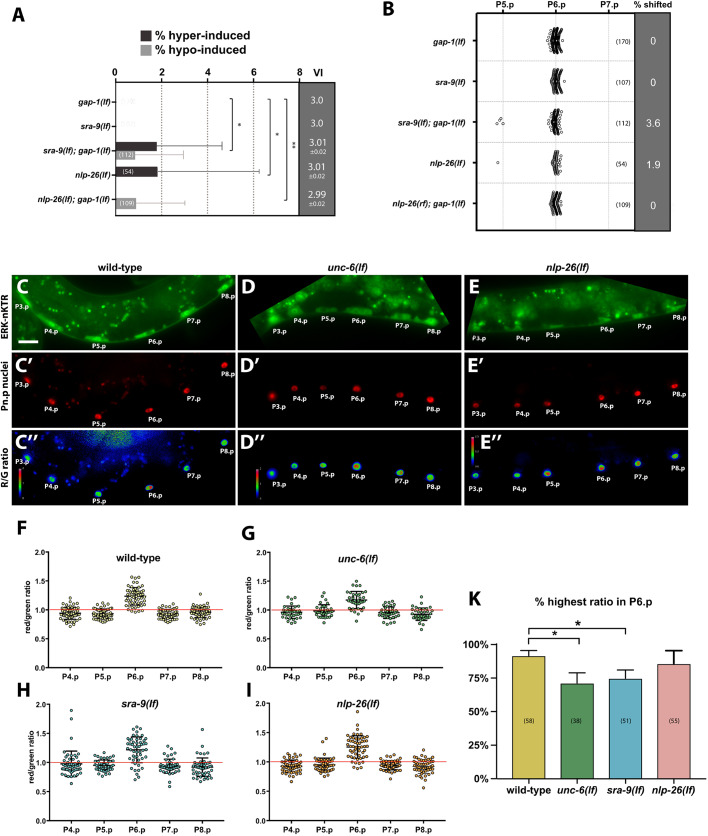
***sra-9(lf)* and *nlp-26(lf)* mutants exhibit an increased error rate in vulval fate specification and higher variability in MPK-1 activation.** (A) Percentage of animals with hyperinduced (dark gray) or hypo-induced (light gray) vulval phenotypes for the indicated genotypes. The gray column to the right shows the VI value (mean±s.e.m.). (B) Animals with 1° cell fate shifts. For each genotype, the percentage of animals showing a 1° fate shift is shown in the gray column to the right. (C-E″) ERK-nKTR biosensor activity in the VPCs of wild-type (C-C″), *unc-6(lf)* (D-D″) and *nlp-26(lf)* (E-E″) mid-L2 larvae. The top row shows the ERK-nKTR::mClover signal (green), the middle row the mCherry::H2B reference (red) and the bottom row the red/green ratio images. Note in E″ the relatively higher activity in P5.p than in P6.p. (F-I) ERK-nKTR biosensor activity levels plotted as red/green intensity ratios in the VPCs of wild-type (F), *unc-6(lf)* (G), *sra-9(lf)* (H) and *nlp-26(lf)* (I) mid-L2 larvae. (K) Percentages of animals exhibiting highest biosensor activity in P6.p. Error bars show the 95% confidence intervals calculated by bootstrapping with a resampling size of 10,000. Statistical significance was calculated using a *t*-test for independent samples in A and nonparametric, unpaired Mann–Whitney test in K. The numbers in brackets in each plot refer to the numbers of animals scored. *P<0.05, **P<0.01. Scale bar: 5 µm.

Mutants *sra-9(lf)* and *nlp-26(lf)* exhibited hyper- or hypo-induced vulval phenotypes at a low frequency when combined with the *gap-1(lf)* mutation (Fig. 5A). Rarely, even *nlp-26(lf)* single mutants exhibited abnormal vulval induction. In addition, we observed 1° cell fate shifts from P6.p to P5.p in *nlp-26(lf)* single mutants as well as in *sra-9(lf)*; *gap-1(lf)* double mutants (Fig. 5B). Given the increased error rate in vulval induction in *sra-9(lf)* and *nlp-26(lf)* mutants, we examined whether the loss of LIN-3 polarity affects activation of the MAP kinase MPK-1 in VPCs, using an ERK-nKTR biosensor (de la Cova et al., 2017). Briefly, upon phosphorylation by MPK-1 the ERK-nKTR::mClover protein translocates from the nucleus to the cytoplasm of the VPCs. By co-expressing a nuclear mCherry::H2B marker together with the ERK-nKTR::mClover biosensor on a single bi-cistronic mRNA, MPK-1 activity can be quantified as the ratio of the nuclear mCherry::H2B (red) signal intensity divided by the nuclear ERK-nKTR::mClover (green) signal intensity (Fig. 5C-E). For each VPC and animal, the red/green ratio was normalized to the mean of the ratios in P4.p through P8.p in that animal (Fig. 5F-I) (P3.p was not analyzed because it only assumes a VPC fate in around 50% of the animals). In wild-type larvae, MPK-1 activity was highest in P6.p in 91% of the animals, consistent with published data of de la Cova et al. (2017) (Fig. 5F,K). In *unc-6(lf)* mutants, the difference between MPK-1 activity in P6.p and the other VPCs was diminished overall, and the frequency of animals exhibiting highest MPK-1 activity in P6.p was reduced to 71% (Fig. 5G,K). In *sra-9(lf)* and *nlp-26(lf)* mutants, we also measured a greater variability in MPK-1 activity with a significant fraction of animals showing average or below average MPK-1 activity in P6.p (Fig. 5H-K).

Thus, the robust MPK-1 activation observed in P6.p of wild-type larvae is perturbed by global AC depolarization in *unc-6(lf)* mutants or by specific loss of LIN-3 polarity in *sra-9(lf)* or *nlp-26(lf)* mutants. This increased variability in MPK-1 activity after the loss of LIN-3 polarity in the AC may underlie the elevated error rate in vulval induction.

### LIN-3 polarity is necessary for precise AC to P6.p alignment

One mechanism for ensuring robust vulval induction involves the progressive alignment of P6.p with the AC during the L2 stage (Grimbert et al., 2016). Despite initial variability in the location of the AC relative to P6.p in wild-type early L2 larvae, the AC and P6.p are precisely aligned to each other by the early L3 stage. LIN-3 signaling is required for the migration of VPCs towards the AC (Grimbert et al., 2016). Together with lateral LIN-12 NOTCH signaling between the VPCs, which compete for the inductive signal, this results in an almost invariant alignment of the AC with the nearest VPC P6.p (Huelsz-Prince and van Zon, 2017).

**Fig. 6.**
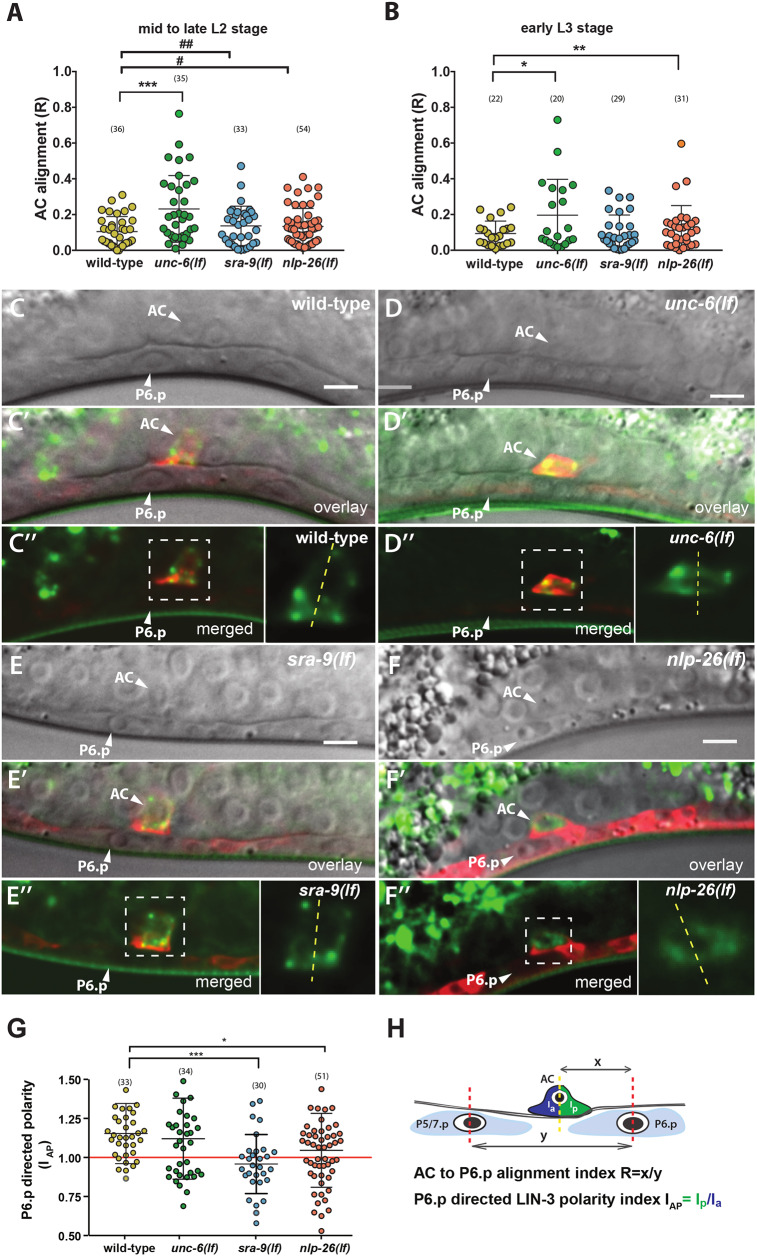
**SRA-9 and NLP-26 are required for efficient AC to P6.p alignment and P6.p-directed LIN-3 polarity.** (A,B) AC to P6.p alignment indices *R* in mid- to late L2 (A) and early L3 (B) larvae of the indicated genotypes. A *t*-test for independent samples and equal variance was used to determine statistical significance between the wild-type and mutants at each developmental stage. # indicates the results of an *F*-test for variance, showing differences in the range of the measured values. (C-C″) Nomarski images of the mid-sagittal plane (C), overlaid with summed z-projections of the *P_cdh3_::mCherry::PLCδ^PH^* (red) and the *mNGr::lin-3* (green) signals (C′) and merged *z*-projections (C″) in the wild-type. (D-F″) Images as for C-C″, but taken in *unc-6(lf)* (D-D″), *sra-9(lf)* (E-E″) and *nlp-26(lf)* (F-F″) mid-L2 larvae. The AC and P6.p nuclei are labeled with arrowheads. The insets in (C″,D″,E″,F″) show approximately 2.5-fold magnifications of the ACs in the regions outlined by the dashed boxes. The dashed yellow lines in the insets indicate the border between the anterior and posterior halves of the AC that were measured to calculate I_AP_, as illustrated in H. Note the enrichment of mNGr::LIN-3 in the anterior half of the AC facing P6.p. in the wild-type (C″) and the *unc-6(lf)* mutant (D″) In the AC of the *sra-9(lf)* (E″) and *nlp-26(lf)* (F″) mutants, mNGr::LIN-3 is more uniformly distributed along the anterior-posterior axis. (G) I_AP_ values for animals of the indicated genotypes with *R*>0.09 (i.e. before complete AC to P6.p alignment, when P6.p-directed secretion can be observed). Statistical significance was calculated using a *t*-test for independent samples. (H) Scheme of the measurements taken to calculate the relative AC to P6.p alignment index (*R*) and the P6.p-directed LIN-3 polarity index (I_AP_). ^#^*P*<0.05, ^##^*P*<0.01; *P<0.05, **P<0.01, ***P<0.001. Scale bars: 5 µm.

We thus investigated whether changes in LIN-3 polarity or in global AC polarity affect the AC to P6.p alignment. For this purpose, we measured the distance between the AC and P6.p, as well as the distance between P6.p and P5.p (or P7.p if the AC was located posterior to P6.p) and calculated the relative P6.p to AC alignment index *R* (Fig. 6A,B,H). In wild-type mid- to late L2 and early L3 larvae, *R* never exceeded a value of 0.4, signifying that the AC was always situated closest to P6.p. In 18% (*n*=35) of *unc-6(lf)* L2 larvae, *R* exceeded a value of 0.4, and the AC was occasionally situated equidistant or even closer to P5.p or P7.p (Fig. 6A). By the early L3 stage, the AC in *unc-6(lf)* mutants had aligned with P6.p in 90% of the cases (Fig. 6B). In *nlp-26(lf)* and *sra-9(lf)* mutants, the defects in AC to P6.p alignment were more subtle. The mean alignment index *R* was slightly higher and the variability (i.e. variance) of *R* was increased in *sra-9(lf)* and *nlp-26(lf)* larvae at the mid- to late L2 stage compared with wild-type larvae (Fig. 6A). By the early L3 stage, the AC and P6.p were properly aligned in all *sra-9(lf)* and most *nlp-26(lf)* larvae (Fig. 6B).

We conclude that polarized localization of LIN-3 in the AC is not absolutely required for AC to P6.p alignment, but it increases the fidelity of the process. The increased error rate in vulval induction and the occasional 1° cell fate shifts in *unc-6(lf)*, *sra-9(lf)* and *nlp-26(lf)* mutants may be caused by the less precise AC to P6.p alignment.

### LIN-3 polarity is directed towards P6.p

In the course of our analysis, we noticed that the mNGr::LIN-3 distribution in the AC was not only polarized along the dorso-ventral axis, but also biased along the anterior-posterior axis towards P6.p before the AC and P6.p had completely aligned (Fig. 6C). We thus measured the LIN-3 polarity along the anterior-posterior axis, applying the same method as described above for the dorso-ventral polarity index to calculate a P6.p-directed anterior-posterior LIN-3 polarity index I_AP_ (Fig. 6H). An index I_AP_>1 indicates that LIN-3 distribution is biased towards P6.p. We measured I_AP_ in wild-type, *unc-6(lf)*, *sra-9(lf)* and *nlp-26(lf)* mutants between the mid-L2 and early L3 stages, before P6.p and the AC had fully aligned (i.e. in animals with *R*>0.09). In wild-type larvae, the mean I_AP_ was significantly higher than 1 (*P*<0.0001), indicating that LIN-3 distribution in the AC is directed towards P6.p (Fig. 6C,G). In *sra-9(lf)* and *nlp-26(lf)* mutants, the mean I_AP_ was reduced and did not significantly differ from 1 [*P*=0.23 for *sra-9(lf)* and *P*=0.16 for *nlp-26(lf)*]. Thus, P6.p-directed LIN-3 polarity is lost in *sra-9(lf)* and *nlp-26(lf)* mutants (Fig. 6E-G). Interestingly, the mean I_AP_ in *unc-6(lf)* mutants was significantly higher than 1 (*P*<0.01) and comparable with the wild-type I_AP_ (Fig. 6D,G). Thus, the P6.p-directed LIN-3 polarity in the AC does not depend on *unc-6*. We also tested whether the relative distance *R* between the AC and P6.p correlated with the P6.p-directed LIN-3 polarity I_AP_. (In those cases where the AC aligned with P5.p or P7.p, we quantified I_AP_ and *R* relative to these VPCs.) In wild-type larvae, we observed a tendency to increased P6.p-directed LIN-3 polarity I_AP_ with decreased AC to P6.p distance *R* (Fig. S5A). Thus, as P6.p approached the AC LIN-3, localization became more polarized towards P6.p. This trend was weaker in *unc-6(lf)* and absent in *sra-9(lf)* and *nlp-26(lf)* mutants (Fig. S5B-D).

Our data thus far suggest that during the process of AC alignment, the AC senses the proximity of the nearest VPC and progressively channels LIN-3 secretion into this direction. The perception of the nearest VPC appears to be preserved in *unc-6(lf)* mutants, although the overall efficiency is slightly reduced as a result of loss of dorso-ventral AC polarity. On the other hand, NLP-26 and SRA-9 are required, directly or indirectly, for the communication between the AC and the most proximal VPC in order to induce the asymmetric distribution of LIN-3.

### *nlp-26* is expressed and acts in the VPCs whereas *sra-9* functions in the AC

**Fig. 7.**
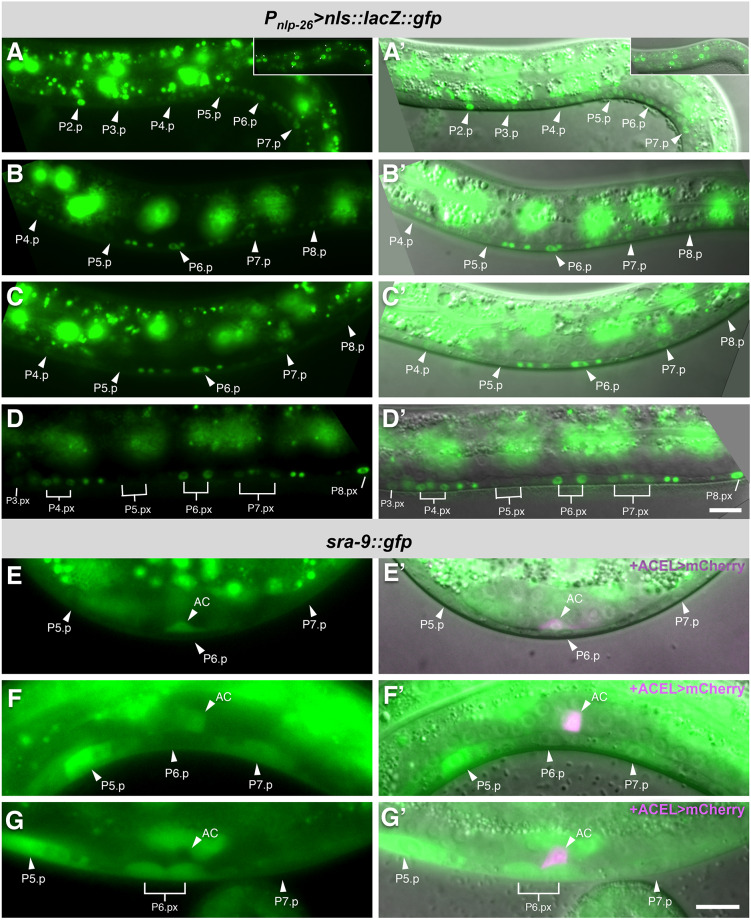
***nlp-26* is expressed in the VPCs and *sra-9* in the AC of L2 larvae.** (A,A′) *P_nlp-26_*>*nls::lacZ::gfp* reporter expression in the VPCs and Pn.a neurons of an early L2 larva (A) and overlay with the corresponding Nomarski image (A′). The insets in the upper right show a top layer with some of the hyp7 nuclei expressing the *P_nlp-26_*-*nls::lacZ::gfp* reporter. (B-C′) Expression in mid-L2 (B,B′) and late L2/early L3 larvae (C,C′). (D,D′) *nlp-26* reporter expression in the VPC descendants at the Pn.px stage in a late L3 larva. Note the relatively strong expression in the 3° P4.p and P8.p descendants after they had fused with hyp7. (E,E′) Endogenous SRA-9::GFP expression in the AC of a mid-L2 larva (E), merged with the AC marker *zhIs127[ACEL>mCherry]* in magenta and overlaid on the corresponding Nomarski image (E′). (F-G′) SRA-9::GFP expression in the AC as well as in P5.p and P7.p at the early L3 stage (F,F′) and in P6.px cells after the first round of VPC divisions at the mid-L3 stage (G,G′). No more AC expression of SRA-9::GFP was detected from the mid-L3 stage on. The arrowheads point at the VPC and AC nuclei and the lines in D,G at the VPC descendants. Scale bars: 10 µm.

Because *nlp-26* encodes a predicted secreted neuropeptide-like protein, we hypothesized that NLP-26 may constitute a signal secreted by the VPCs or the VNC. The *gfp::nlp-26(zh113)* strain, which carries a *gfp* insertion in place of the signal sequence, did not show any GFP expression, probably because endogenous *nlp-26* expression levels are very low. We thus generated a multicopy transcriptional *nlp-26* reporter by fusing 1.4 kb of the *nlp-26* 5′ regulatory region to an *nls::lacZ::gfp* reporter cassette in order to confine the GFP signal to the nuclei (*zhEx632[P_nlp-26_*-*nls::lacZ::gfp]*). This transcriptional *nlp-26* reporter was dynamically expressed in the VPCs and their sister Pn.a neurons in the VNC. In early to mid-L2 larvae, *P_nlp-26_*-*nls::lacZ::gfp* was expressed in all VPCs and their sister Pn.a neurons (Fig. 7A). In addition, *nlp-26* was strongly expressed in the hyp7 cell at all stages (Fig. 7A, inset). By the late L2/early L3 stage, *nlp-26* transcription was upregulated in P6.p and the P6.a neurons, while expression faded in the other VPCs (Fig. 7B,C). *nlp-26* continued to be expressed in the P6.px daughter cells of mid-L3 larvae (Fig. 7D). Note that descendants of the 3° VPCs P3.p, P4.p and P8.p began to express *nlp-26* after they had fused with hyp7 (Fig. 7D).

To observe *sra-9* expression, we inserted by CRISPR/Cas9-mediated genome editing a *gfp::3×Flag* cassette in frame at the 3′-end of the *sra-9* coding sequences to generate the endogenous *sra-9* reporter (*zh151[sra-9::gfp::loxP::3×Flag]*). In mid-L2 larvae, SRA-9::GFP was expressed in the AC and other uterine cells but not in the VPCs or the VNC (Fig. 7E). From the early L3 stage on, SRA-9::GFP expression was also observed in the VPCs, whereas AC expression faded until it was absent in mid-L3 (Pn.px stage) larvae (Fig. 7F,G).

**Fig. 8.**
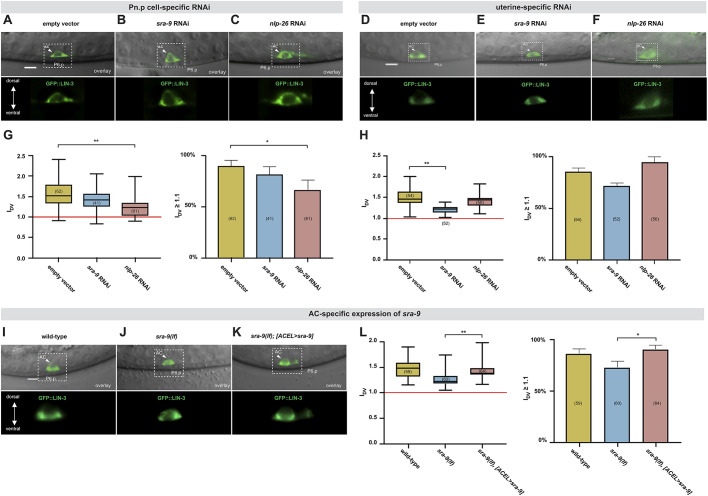
***nlp-26* acts in the Pn.p cells and *sra-9* in the AC.** (A-C) Top rows: Nomarski images of a mid-sagittal plane overlaid with the GFP::LIN-3 reporter expression (green) after Pn.p cell-specific RNAi in an empty vector control (A), *sra-9* RNAi (B) and *nlp-26* RNAi (C) animals at the mid-L2 stage. (D-F) GFP::LIN-3 reporter expression after uterine-specific RNAi in an empty vector control (D), *sra-9* RNAi (E) and *nlp-26* RNAi (F) animals at the mid-L2 stage. The position of the AC (arrowheads) and the P6.p nuclei are indicated. The dashed boxes outline the AC regions magnified about 2.5-fold in the bottom row. (G,H) Box plots of the LIN-3 polarity index I_DV_ after Pn.p cell-specific RNAi (G) and after uterine-specific RNAi (H), quantified at the mid-L2 stage. The bar graphs to the right of each box plot show the percentages of animals with a LIN-3 I_DV_>1.1. (I-K) Top rows: Nomarski images of a mid-sagittal plane overlaid with the GFP::LIN-3 (green) reporter signal in a wild-type control (I), a *sra-9(zh108lf)* mutant (J) and a *sra-9(zh108lf)* mutant carrying the AC-specific *zhIs143[P_ACEL_>sra-9::SL2::mCherry]* rescue construct (K). (L) Box plot of the LIN-3 I_DV_ in the wild-type, *sra-9(zh108lf)* and *sra-9(zh108lf); zhIs143[P_ACEL_>sra-9::SL2::mCherry]* background. The bar graph to the right shows the percentages of animals with a LIN-3 I_DV_>1.1. Error bars in the box plots indicate the minimum and maximum values. Statistical significance was calculated with a *t*-test for independent samples of unequal variance. Error bars in the bar graphs indicate the 95% confidence intervals. For the bar graphs, statistical significance was calculated using the nonparametric, unpaired Mann–Whitney test. The numbers in brackets refer to the numbers of animals scored for each condition. *P<0.05, **P<0.01. Scale bars: 5 μm.

The *nlp-26* and *sra-9* expression patterns suggest that NLP-26 might act as a signal secreted by the VPC that polarizes LIN-3 trafficking in the AC, whereas SRA-9 could function in the AC to transduce the VPC signal. We therefore performed Pn.p cell- and uterine-specific RNAi of *nlp-26* and *sra-9* using the *zhEx418[lin-31::rde-1]* (Haag et al., 2014) and *qyIs102[fos-1ap::rde-1]* (Matus et al., 2010) transgenes, respectively, in the *rrf-3(lf); rde-1(lf)* background, as described in Yang et al. (2017) (Fig. S6A,B). We then quantified the polarity of the GFP::LIN-3 reporter in the AC under the different RNAi conditions. Pn.p cell-specific *sra-9* RNAi had no significant effect on GFP::LIN-3 polarity compared with empty vector controls (Fig. 8A,B,G), but *nlp-26* RNAi reduced GFP::LIN-3 polarity (Fig. 8C,G). Conversely, uterine-specific *sra-9* RNAi reduced LIN-3 polarity, whereas *nlp-26* RNAi had no significant effect compared with empty vector controls (Fig. 8D-F,H). To further define the cellular focus of *sra-9* in the uterus, we expressed wild-type *sra-9* under control of the *lin-3* AC-specific element (Hwang and Sternberg, 2004) together with an mCherry reporter in the *sra-9(lf)* background, using the bi-cistronic transgene *zhIs143[P_ACEL_>sra-9::SL2::mCherry]* (Fig. S6C). AC-specific expression of *sra-9* rescued the GFP::LIN-3 polarity defects of *sra-9(lf)* mutants, suggesting that SRA-9 functions in the AC (Fig. 8I-L). Thus, NLP-26 may act as a feedback signal secreted by the VPCs to polarize, via SRA-9, the secretion of LIN-3 by the AC.

## DISCUSSION

**Fig. 9.**
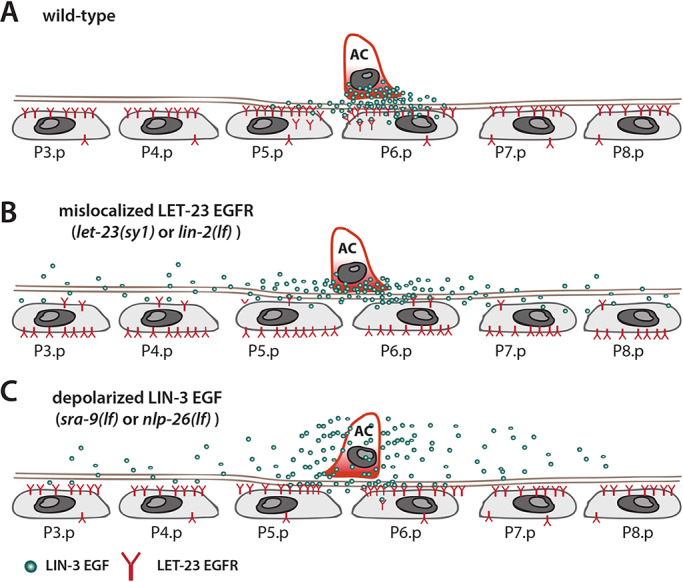
**Model for polarized LIN-3 secretion.** (A) In the wild-type, the limiting amounts of LIN-3 secreted by the AC are efficiently sequestered by an excess of LET-23 on the basolateral side of P6.p to prevent spreading of the signal to distal VPCs. (B) The *let-23(sy1)* mutation causes the mislocalization of LET-23 from the basolateral to the apical side of the VPCs. Therefore, LIN-3 is no longer sequestered by LET-23 in P6.p and the inductive signal can spread to the distal VPCs. The distal VPCs are induced to differentiate if the RAS/MAPK is simultaneously hyperactivated, for example by the *gap-1(lf)* mutation. (C) De-polarization of LIN-3 in the AC, for example in *sra-9(lf)* or *nlp-26(lf)* mutants, likewise results in reduced ligand sequestering by P6.p and the spreading of LIN-3 to more distal VPCs*.*

*C. elegans* vulval cell fate specification serves as an excellent *in vivo* model to analyze the subcellular localization of the EGF ligand and receptor at single cell resolution. It has been proposed that the limiting amounts of LIN-3 secreted by the AC form a gradient that can act in a dose-dependent manner to specify the different fates of the proximal VPCs (Katz et al., 1995). Moreover, LET-23 expressed on the basolateral surface of the nearest VPC (P6.p) sequesters most of the LIN-3 signal (Hajnal et al., 1997) (Fig. 9A). If LET-23 is mislocalized to the apical VPC compartment, then less LIN-3 is sequestered by P6.p and the inductive signal can reach the distal VPCs (Fig. 9B). Thus, LET-23 localized on the basolateral membrane of P6.p limits the range of the graded LIN-3 signal.

### Polarized LIN-3 secretion determines the distribution of the inductive signal

Here, we show that both LET-23 localization in the VPCs and polarized secretion of LIN-3 by the AC regulate the range of the inductive signal (Fig. 9C). Our results indicate that the AC polarizes LIN-3 trafficking from the endoplasmic reticulum towards its ventral side that faces the VPCs. This polarized secretion depends on the global AC polarity established by the ventral to dorsal UNC-6 Netrin gradient (Ziel et al., 2009). LIN-3 secretion is then further enriched in the direction of the nearest VPC, usually P6.p, along the anterior-posterior axis. Although dorso-ventral LIN-3 polarity is strongly reduced in *unc-6 netrin(lf)* mutants, the secretion of LIN-3 in the direction of the nearest VPC along the anterior-posterior axis occurs independently of the UNC-6-dependent AC polarity.

Using an unbiased, genome-wide approach, we identified two genes, *sra-9* and *nlp-26*, that are specifically required for polarized LIN-3 localization and do not affect global AC polarity: *sra-9* encodes an orphan GPCR protein and *nlp-26* a secreted neuropeptide-like protein. *nlp-26* is expressed in the VNC neurons and the VPCs, where it shows a dynamic expression pattern until the late L2/early L3 stage, when expression becomes upregulated in the 1° VPC that sequesters the LIN-3 signal. Thus, NLP-26 could act as a secreted cue from the VPCs that polarizes LIN-3 trafficking in the AC. This hypothesis is supported by the finding that Pn.p cell-specific depletion of *nlp-26*, but not of *sra-9*, reduced LIN-3 polarity in the AC. An endogenous *sra-9* reporter was expressed in the AC of mid-L2 larvae, the period when LIN-3 polarity is first observed, and AC-specific expression of *sra-9* rescued the LIN-3 polarity defects of *sra-9(lf)* mutants. Thus, SRA-9 could be required for the transduction of the NLP-26 signal in the AC. Whether SRA-9 functions as a receptor for NLP-26 in the AC remains to be determined. Taken together, SRA-9 and NLP-26 appear to be part of a sensing mechanism used by the AC to detect the location of the nearest VPC and focus LIN-3 secretion into the direction of the future 1° VPC. Because VPCs migrate towards the AC along a LIN-3 gradient while competing for the inductive AC signal via DELTA/NOTCH-mediated lateral inhibition, AC to P6.p alignment and 1° vulval cell fate specification are tightly coupled (Huelsz-Prince and van Zon, 2017). Our data indicate that the AC plays an active part in this feedback mechanism. We propose that the AC directs and channels LIN-3 secretion towards the ‘leader’ in the race of the VPCs for the 1° cell fate (Fig. 9A). As a result, the LIN-3 gradient becomes progressively restricted and the closest VPC (the ‘winner’) receives most of the inductive signal.

### Polarized LIN-3 secretion ensures robust vulval induction

Vulval fate specification is extremely robust. In the wild-type larvae grown under standard conditions, P6.p adopts the 1° fate in 99.9% of the cases and always induces the 2° fate in its neighbors P5.p and P7.p (Braendle and Félix, 2008). Previous studies have shown that this robustness is achieved through cross-talk between the inductive EGFR/RAS/MAPK and the lateral DELTA/ NOTCH signaling pathways, which results in the almost invariable alignment of the 1° vulval cell P6.p with the AC (Barkoulas et al., 2013; Berset et al., 2001; Grimbert et al., 2016; Yoo et al., 2004). Here, we show that the polarized and directed secretion of LIN-3 by the AC is an additional mechanism that ensures robust vulval induction. Even though *nlp-26(lf)* and *sra-9(lf)* mutants do not exhibit penetrant vulval phenotypes, they do display (at a low penetrance) errors in fate patterning, shifts in 1° fate selection and a less precise AC to P6.p alignment. All these defects are probably due to increased variability in MAPK activation in P6.p, as observed using a MPK-1 biosensor. Thus, LIN-3 secretion by the AC is an actively regulated process that depends on the global AC polarity determined by the ventral to dorsal UNC-6 Netrin gradient and on a sensing mechanism to detect the nearest VPC, which is mediated by the neuropeptide-like protein NLP-26 with the GPCR SRA-9 as a candidate receptor. Whether SRA-9 and NLP-26 regulate the secretion of other proteins by the AC in addition to LIN-3 remains to be determined. Moreover, *nlp-26(lf)* and *sra-9(lf)* may act in other tissues besides the VPCs and AC, respectively, and could also affect AC alignment and vulval induction indirectly.

In summary, the directed secretion of a growth factor towards the target tissue may be a common strategy to achieve the robust and efficient patterning of cell fates. In mammalian cells also, the confined subcellular localization of EGF family ligands regulates the range and directionality of their signaling activity. For example, TGFα is sorted to the basolateral membrane compartment of epithelial MDCK cells to restrict signal diffusion and promote juxtacrine signaling (Singh and Coffey, 2014). Similarly, Pro-Epiregulin is sorted to the basolateral compartment of MDCK cells and its mislocalization to the apical compartment causes hyperproliferative, locally invasive tumors (Singh et al., 2013). It will be interesting to investigate whether similar feedback mechanisms between signal sending and signal receiving cells are used in the EGF signaling pathway of other species.

## MATERIALS AND METHODS

### General methods and strains used

Unless noted otherwise, *C. elegans* strains were maintained at 20°C on NGM (Nematode Growth Medium) plates applying standard methods (Brenner, 1974). The *C. elegans* Bristol variety N2 was used as wild-type reference, and strains used for the experiments were derivates of N2. The following alleles and transgenes were used: LGI: *unc-40(e271)* (Hedgecock et al., 1990), *zhIs127[P_ACEL Δpes10_*>*mCherry, unc-119(+)]*; LGII: *qyIs23[P_cdh-3_::PLC∂PH::mCherry, unc-119(+)]* (Ziel et al., 2009), *arTi85[P_lin-31_::ERK-KTR(NLS3)-mClover-T2A-mCherry-H2B::unc-54 3*′*UTR, rps-27p::NeoR::unc-54 3*′*UTR]* (de la Cova et al., 2017), *rrf-3(pk1426)* (Simmer et al., 2002), *let-23(sy1)* (Katz et al., 1996), ttTi5605 (Frøkjær-Jensen et al., 2008), *sra-9(zh108*) (this study) and *sra-9(zh151[sra-9::gfp::loxP::3×Flag])* (this study); LGIII: *unc-119(ed3), madd-2(tr103)* (Alexander et al., 2010), *oxTi444, zhIs143[P_ACEL Δpes10_>sra-9_genomic_::SL2::mCherry::unc-54 3′UTR]* and *unc-119(+)* (this study); LGIV: *lin-3(e1417)* (Hwang and Sternberg, 2004) and *lin-3(zh112[mNGr::loxP::3×Flag])*; LGV: *nlp-26(zh113[gfp::loxP::3×Flag])* (this study) and *rde-1(ne219)*; LGX: *unc-6(ev400)* (Hedgecock et al., 1990) and *gap-1(ga133)* (Hajnal et al., 1997); unknown LG: *zhIs67[gfp::lin-3, unc-119(+)]* (this study) and *qyIs102[fos-1ap::rde-1;myo2::yfp; unc-119]* (Matus et al., 2010). Extrachromosomal arrays used were *zhEx418[lin-31::rde-1, myo2-mcherry]* (Haag et al., 2014), *zhEx632[P_nlp-26_-nls::lacZ::gfp, myo2-mcherry]* (this study) and *zhEx668.1 to zhEx668.3[P_cdh-3_::unc-40_minigene_::gfp::unc-54 3′UTR]* (this study).

### Plasmid construction

DNA fragments were isolated by PCR using templates and primers described in the supplementary Materials and Methods and assembled using Gibson recombinase (Gibson et al., 2009) or by ligation of restriction fragments.

### CRISPR/CAS9-generated alleles

#### mNGr::lin-3(zh112)

To insert the *mNGr::3×Flag* sequence in the 5′ region of the *lin-3* locus, the CRISPR/Cas9 system according to Dickinson et al. (2015) was applied. The repair template plasmid pLM5 was injected at a concentration of 8 ng/μl, the two single guides with integrated CAS9 plasmids pLM12 and pLM13 at a concentration of 40 ng/μl together with the recommended co-injection markers pGH8 (Addgene 19359) at 10 ng/μl, pCFJ104 (Addgene 19328) at 5 ng/μl and pCFJ90 (Addgene 19327) at 2.5 ng/μl.

#### gfp::nlp-26(zh113)

To insert the *gfp::3×Flag* sequence in the 5′ region of the *nlp-26* locus, the repair template plasmid pLM7 was injected at a concentration of 10 ng/μl, the three single guides with integrated CAS9 plasmids pLM8, pLM9 and pLM10 at a concentration of 50 ng/μl together with the same co-injection markers used for *zh112*.

#### sra-9(zh108)

This deletion allele was generated according to the Arribere et al. (2014) protocol, with the following single guide sequences: TTG GCA AAG TTC TAG TTA T, AC C AAT TGA ATT GCT GGA T.

#### sra-9::gfp(zh151)

To insert a *gfp::3×Flag* cassette into the 3′ region of the *sra-9* locus, the repair template plasmid pSS22 was injected at a concentration of 10 ng/μl, the two single guides with integrated CAS9 plasmids pSS20 and pSS21 at a concentration of 50 ng/μl together with the same co-injection markers used for *zh112*.

Selection of homologous integrands was carried out according to the protocol of Dickinson et al. (2015).

### RNA interference

RNAi was performed using the feeding method as described by Kamath and Ahringer (2003). P0 worms were synchronized at the L1 stage, transferred to NGM plates containing 3 mM IPTG and 50 ng/ml ampicillin seeded with the indicated RNAi bacteria and allowed to grow for 5-7 days at 20°C, after which the F1 progeny were analyzed. For Pn.p cell-specific RNAi, a strain of the genotype *rrf-3(pk1426)II; unc-119(ed4)III; rde-1(ne219)V; zhEx418[lin-31::rde-1; myo2-mcherry]* including the *zhIs67[gfp::lin-3, unc-119(+)]* reporter was used and LIN-3 polarity was measured in the F1 generation in three independent experiments. To control the tissue specificity of this RNAi strain, we performed global and Pn.p cell-specific *lin-3* RNAi and observed expression of the *zhIs67* reporter (Fig. S6). For uterine-specific RNAi, a strain of the genotype *rrf-3(pk1426)II; unc-119(ed4)III; rde-1(ne219)V; qyIs102[fos-1ap::rde-1; myo-2::yfp]* including the *zhIs67[gfp::lin-3, unc-119(+)]* reporter was used and LIN-3 polarity was measured in the F1 generation in three independent experiments.

### Microscopy and image analysis

Images were acquired using an Olympus BX61 wide-field microscope equipped with a Cr.E.S.T. X-light spinning disc system, a Lumencor SPECTRA X light engine and a Hamamatsu Orca CMOS camera or an iXon Ultra 888 EMCCD camera controlled by the Visitron VisiView 2.1.1 software. Fluorescent image *z*-stacks of the mNGr::LIN-3 and GFP::LIN-3 reporters were processed using the Huygens Deconvolution software (SVI, Center for Microscopy and Image Analysis, University of Zürich). Images were analyzed with Fiji/ImageJ software (Schindelin et al., 2012).

### Determination of the developmental stage

The developmental stages were determined by measuring the gonad length in Nomarski images, as described (Kimble and Hirsh, 1979). Animals were divided into three groups: early to mid-L2 stage (gonad 30-70 µm), late L2 stage (gonad 70-110 µm) and early to mid-L3 stage (gonad 110-150 µm).

### AC polarity measurements

The dorso-ventral and anterior-posterior AC polarity for LIN-3 and PIP_2_ was measured using a semi-automated script in Fiji, creating summed *z*-projections of image stacks across the AC with 0.2-0.13 μm *z*-spacing with prior deconvolution (for LIN-3::mNGr and GFP::LIN-3) or in which the background had been subtracted (for *P_cdh3_::mCherry::PLCδ^PH^*). A threshold was applied to set pixel intensities outside of the AC to zero. The dorso-ventral polarity index I_DV_ was calculated by dividing the AC into equal areas of the ventral and dorsal halves defined by the center of the AC nucleus in the DIC image and dividing the average ventral signal intensity by the average dorsal intensity. Thus, an I_DV_>1 indicates a higher signal intensity in the ventral half of the AC. An analogous method was used to calculate the P6.p-directed LIN-3 polarity index I_AP_ by dividing the AC into equal halves proximal and distal to P6.p or to the nearest VPC, as shown in Fig. 6H.

### Vulval induction counts

Vulval induction was scored by examining worms at the L4 stage under Nomarski optics as described (Sternberg and Horvitz, 1986). The number of VPCs that had adopted a 1° or 2° vulval cell fate was counted for each animal, and the vulval induction index (VI) was calculated by dividing the total number of induced cells by the number of animals scored. Animals with VI>3 were scored as hyperinduced, and animals with VI<3 as hypo-induced. The different strain combinations compared in the vulval induction assays were generated from progeny obtained in the same crosses.

### AC to P6.p alignment

For the alignment quantification, the distance between the AC and either P6.p or the closest VPC was measured as illustrated in Fig. 6H. To calculate the relative alignment index *R*, this value was divided by the P6.p-P5.p or to P7.p-distance depending on the AC location.

### ERK-nKTR biosensor quantification

MPK-1 activity in the VPCs was measured using the recently established ERK-nKTR biosensor *arTi85*, which is based on the MPK-1 activity-dependent nuclear export of the biosensor (de la Cova et al., 2017). Custom-made ImageJ (Schindelin et al., 2012) and Cell Profiler (Carpenter et al., 2006) scripts were used to process and quantify the images taken in the indicated mutant backgrounds under standardized illumination conditions, as described by Maxeiner et al. (2019). Flat field illumination and background corrections were carried out using blank and dark field images, respectively, taken for each experiment. The nuclear red/green (mCherry::H2B/nKTR::mClover) average intensity ratios were measured in each VPC (except for P3.p) in summed *z*-projections of the five central slices taken with a *z*-spacing of 0.13 μm, relative to the focus of the nuclear mCherry::H2B signal. Because we could not assume that P4.p was not affected, the red/green nuclear ratio for each VPC was normalized to the average of the red/green ratios in P4.p to P8.p in the same animal, rather than normalizing to the ratio in P4.p, as described by de la Cova et al. (2017). The normalized values are plotted in Fig. 5F-I.

### Statistical analysis

Statistical analyses for vulval induction were performed by bootstrapping the data with a resample size of 10,000 samples. The standard deviation within the bootstrapped samples was used to estimate the standard error of the mean (s.e.m.) and the 95% confidence interval. Statistical analysis of continuous measures (I_DV_, I_AP_, *R*) was performed using Student's *t*-tests or Fisher's exact tests as indicated in the figure legends.

## Supplementary Material

Supplementary information
