# Supplementary material for: Polarized epidermal growth factor secretion ensures robust vulval cell fate specification in Caenorhabditis elegans
Source: Development. 2020 Jun 4;147(11):dev175760. doi: 10.1242/dev.175760 (PMC7286359; doi:10.1242/dev.175760)
Supplement: Supplementary information [file develop-147-175760-s1.pdf]

Supplementary information

Supplementary Table S1

cellular function	gene	WB identifier	brief description	LIN-3 polarity	AC polarity
**cell adhesion**					
	dig-1	WBGene00000998	immunoglobulin superfamily	P	n.d.
**cell cycle**					
	cdk-4	WBGene00000406	cyclin-dependent serine/threonine protein kinase	P	n.d.
	cki-1	WBGene00000516	cyclin-dependent kinase inhibitor p27	P	n.d.
	cye-1	WBGene00000871	E-type cyclin	n.d.	n.d.
	cdk-11.1	WBGene00015203	cyclin-dependent serine/threonine protein kinase	P	n.d.
	cdk-2	WBGene00019362	cyclin-dependent serine/threonine protein kinase	P	n.d.
**cell signaling**					
	lag-2	WBGene00002246	DSL family NOTCH ligand	P	n.d.
	nlp-26	WBGene00003764	predicted neuropeptide	D	P
	sra-9	WBGene00005035	G-protein coupled receptor	D	P
	srd-36	WBGene00005114	G-protein coupled receptor	P	n.d.
	srh-8	WBGene00005234	G-protein coupled receptor	P	n.d.
	srh-247	WBGene00005453	G-protein coupled receptor	D	P
	unc-40	WBGene00006776	netrin receptor	D	D
	unc-73	WBGene00006805	guanine nucleotide exchange factor similar to Trio	n.d.	n.d.
	madd-2	WBGene00016539	C1 subfamily of tripartite motif (TRIM) protein	D	D
	toe-2	WBGene00016971	DEP domain containing protein	P	n.d.
**cellular iron ion homeostasis**					
	Y45F10D.4	WBGene00012885	iron-sulfur cluster assembly enzyme	P	n.d.
**chromosome organization**					
	dpy-27	WBGene00001086	SMC4 subunit of mitotic condensin	P	n.d.
	his-72	WBGene00001946	H3 histone	P	n.d.
	hmg-3	WBGene00001973	Subunit of the heterodimeric FACT complex	P	n.d.
**immunity**					
	F26F12.5	WBGene00017836	antimicrobial protein	P	
**ion transport**					
	acr-3	WBGene00000043	nicotinic acetylcholine receptor superfamily	P	n.d.
	mca-1	WBGene00003151	plasma membrane Ca2+ ATPases	P	n.d.
**lipid transport**					
	cav-2	WBGene00000302	caveolin	P	n.d.
	acbp-3	WBGene00009818	acyl-CoA-binding binding domain	D	D
**protein modification**					
	usp-48	WBGene00009267	ubiquitin specific peptidase	D	D
	ugt-14	WBGene00019233	UDP glycosyltransferase	P	n.d.
	gei-17	WBGene00001574	SUMO E3 ligase	D	D
	ulp-1	WBGene00006736	SUMO protease	D	D
**regulation of transcription**					
	lin-1	WBGene00002990	Ets-domain transcription factor	P	n.d.
	lin-9	WBGene00002998	novel protein	P	n.d.
	lin-14	WBGene00003003	novel protein	P	n.d.
	mes-6	WBGene00003224	Polycomb-like chromatin repressive complex	P	n.d.
	nhr-103	WBGene00003693	nuclear hromone receptors	P	n.d.
	nhr-115	WBGene00003705	nuclear hromone receptors	P	n.d.
	sdc-2	WBGene00004746	component of dosage compensation complex	P	n.d.
	sem-4	WBGene00004773	zinc-finger transcription factor	P	n.d.
	din-1	WBGene00008549	spen family transcriptional repressor	P	n.d.
	hpo-11	WBGene00010427	nuclear receptor binding protein 2	P	n.d.
**regulation of translation**					
	fbf-2	WBGene00001402	Pumilio and FBF family translational regulator	D	D
	puf-8	WBGene00004244	Pumilio and FBF family translational regulator	n.d.	n.d.
**small molecule metabolic process**					
	cah-4	WBGene00000282	carbonic anhydrase	D	D
	lact-9	WBGene00012890	beta-lactamase domain-containing protein	P	n.d.
	T22F3.3	WBGene00020696	glycogen phosphorylase isozymes	P	n.d.
**unknown functions**					
	C31H5.5	WBGene00007856	novel protein	n.d.	n.d.
	clec-197	WBGene00008202	C-type lectin	P	n.d.
	T18D3.1	WBGene00011820	novel protein	P	n.d.
	Y69H2.3	WBGene00013481	novel protein	P	n.d.
	ZK795.2	WBGene00014082	novel protein	P	n.d.
	T20D4.11	WBGene00020617	novel protein	P	n.d.
	T02B11.9	WBGene00044777	novel protein	D	D

Development • Supplementary information

Table S1. Top 51 candidate genes identified in a whole-genome RNAi screen.

List of 51 genes that reproducibly showed a Muv phenotype in the primary RNAi screen and in four rounds of rescreening. The candidates are grouped into categories of cellular functions. During the re-screening, global AC polarity using the P_{cdh3}*::mCherry::PLCδ*^{PH} (PIP_2) reporter *qyIs23* and LIN-3 polarity using the GFP::LIN-3 reporter *zhIs67* were scored simultaneously. P stands for normally polarized and D for defective polarity. In some cases (n. d.), the polarity could not be determined or PIP_2 polarity was not scored because LIN-3 polarity was normal. The three genes, in which RNAi lead to depolarization of GFP::LIN-3 but had no effect on global AC polarity are highlighted in orange. Previously known regulators of AC polarity and positioning that were identified in the screen are highlighted in green.

Development • Supplementary information

Supplementary Figures

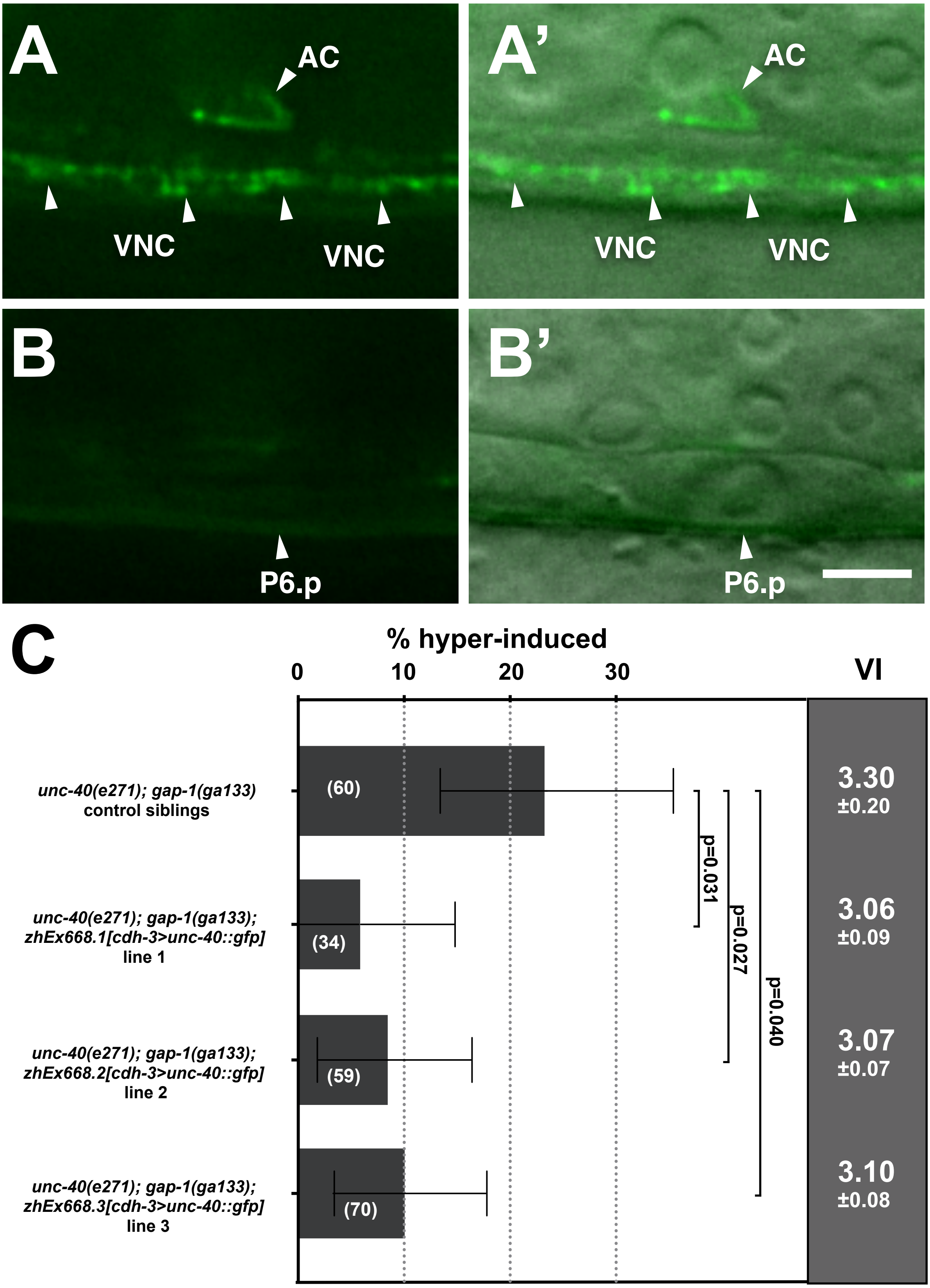

Development • Supplementary information

Figure S1. Expression of *unc-40::gfp* in the AC partially rescues the hyper-induced *unc-40(e271); gap-1(ga133)* phenotype.

(A) Expression of the *cdh-3>unc-40::gfp* transgene in the AC and VNC neurons of an early L3 larva, (A') overlaid on the corresponding Nomarski image. (B,B') show a focal plane of P6.p in the same animal. The scale bar in (B') is 5 μm. (C) The percentages of animals with a hyper-induced vulval phenotype (VI>3) are shown for the three transgenic lines and control siblings lacking the transgene. The gray column to the right shows the average VI ± standard error of the mean. The error bars indicate the 95% confidence intervals calculated by bootstrapping with a resampling size of 10'000. p-values were calculated with a t-test for independent samples. The numbers in brackets in each plot refer to the numbers of animals scored.

Development • Supplementary information

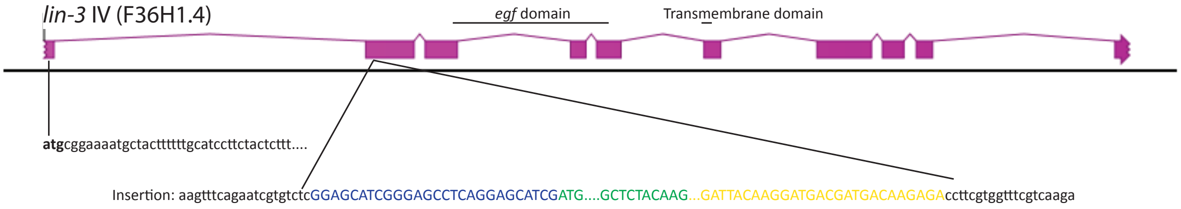

Figure S2. Insertion of the mNGr reporter into the *lin-3* locus (*zh112*).

The sequences are color coded as follows: black: genomic *lin-3* sequences with the initiation codon highlighted in bold; blue: linker sequence; green: mNGr cassette; yellow: 3xFlag tag. In the *zhIs67* strain, a *gfp* cassette was inserted in place of mNGr.

Development • Supplementary information

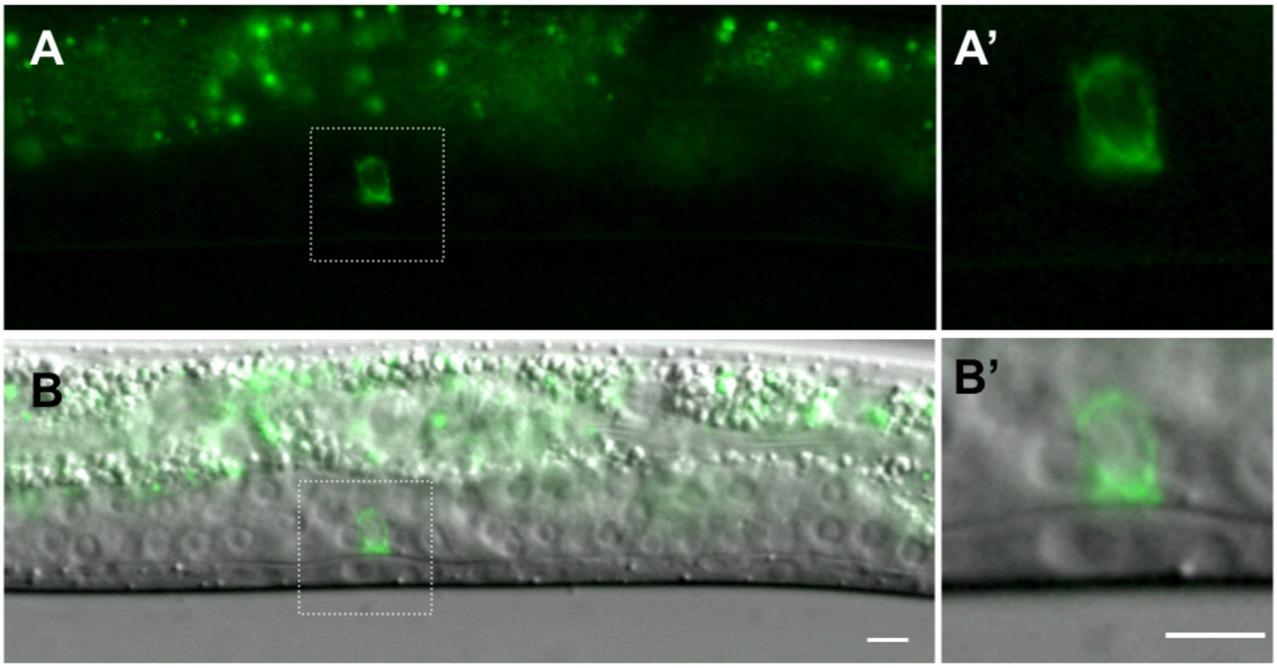

Figure S3. Localization of a multi-copy GFP::LIN-3 transgenic reporter.

(A) Polarized GFP::LIN-3 expression in the AC and (B) merged with the Nomarski image of a late L2 larva using the multi-copy transgene *zhIs67*. The dashed boxes indicate the regions shown enlarged in (A') and (B'). The scale bars are 5 µm.

Development • Supplementary information

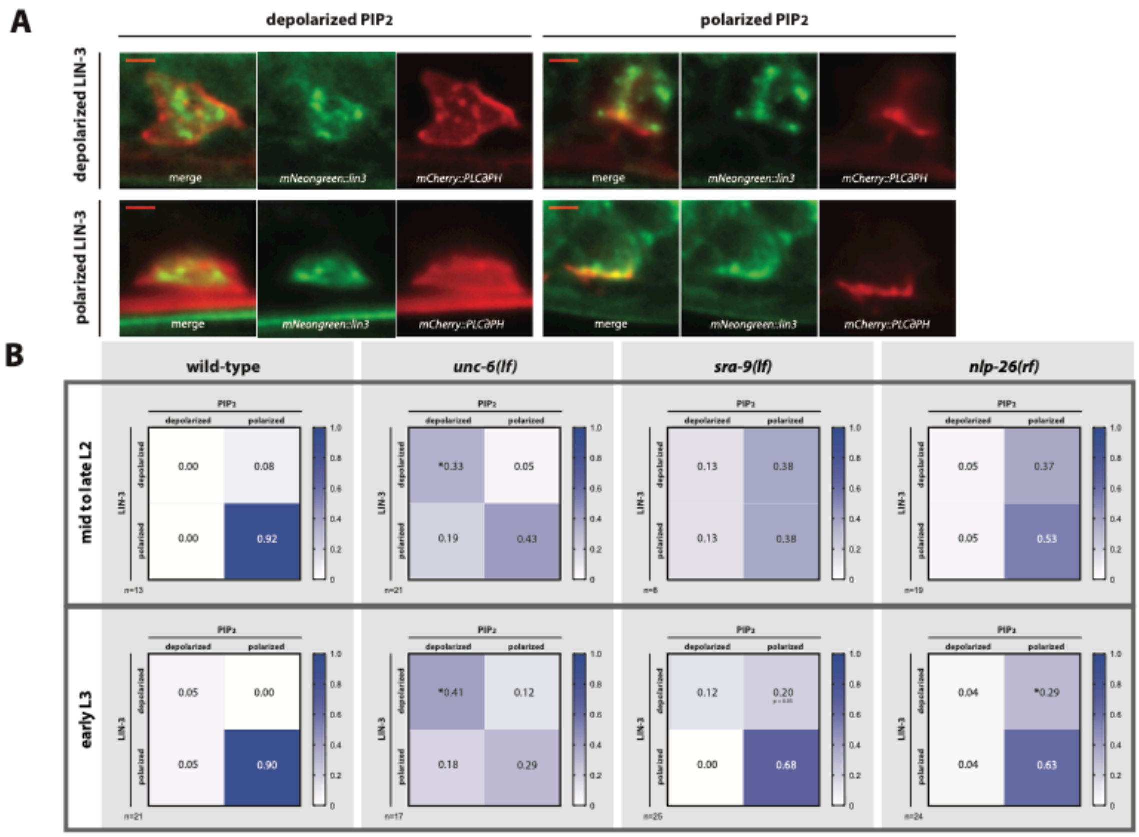

Figure S4. Correlation between global PIP_2 and specific LIN-3 EGF polarity at the single animal level. (A) Examples of summed z-projections of ACs expressing *P_{cdh3}::mCherry::PLCδ^{PH}* PIP_2 marker (red) and the endogenous *mNGr::lin-3* reporter (green) with merged panels to the left depicting the different combinations of polarity observed with the two reporters. The scale bar is 2.5 µm. (B) Heat-maps showing the correlation between LIN-3 and PIP_2 polarity for wild-type, *unc-6(lf), sra-9(zh108)* and *nlp-26(zh113)* mutants at the mid to late L2 and the early L3 larval stages. Sample size n is indicated for each heat map. LIN-3 was scored as polarized for a $I_{DV} > 1.1$. A threshold value of PIP_2 $I_{DV} > 1.2$ was scored as polarized since the mean I_{DV} for the PIP_2 *P_{cdh3}::mCherry::PLCδ^{PH}* reporter in the wild-type was higher than the mean LIN-3 I_{DV}. Statistical significance was calculated with Fisher's exact probability test, ($p < 0.05$ = * and $p < 0.01$ = **).

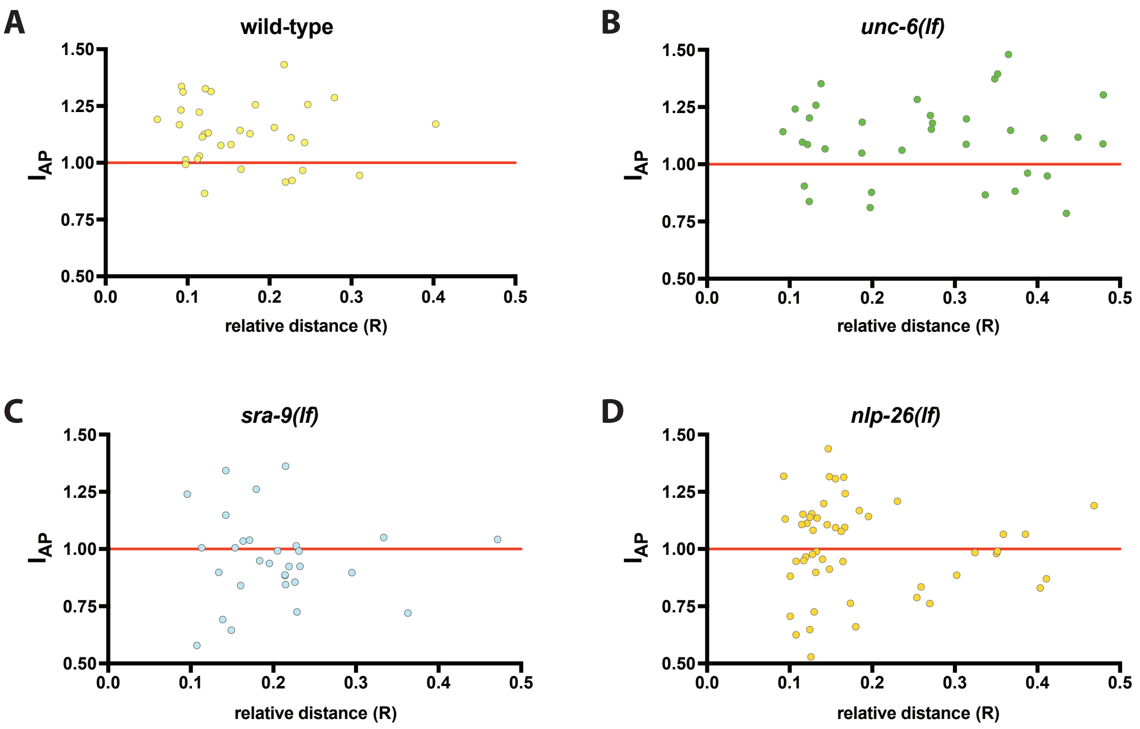

Figure S5. Correlation between the AC to VPC alignment index (R) and VPC-directed LIN-3 polarity (I_{AP}). (A-D) Plots of the VPC-directed LIN-3 polarity index I_{AP} against the relative distance R between AC and the nearest VPC for the indicated genotypes.

Development • Supplementary information

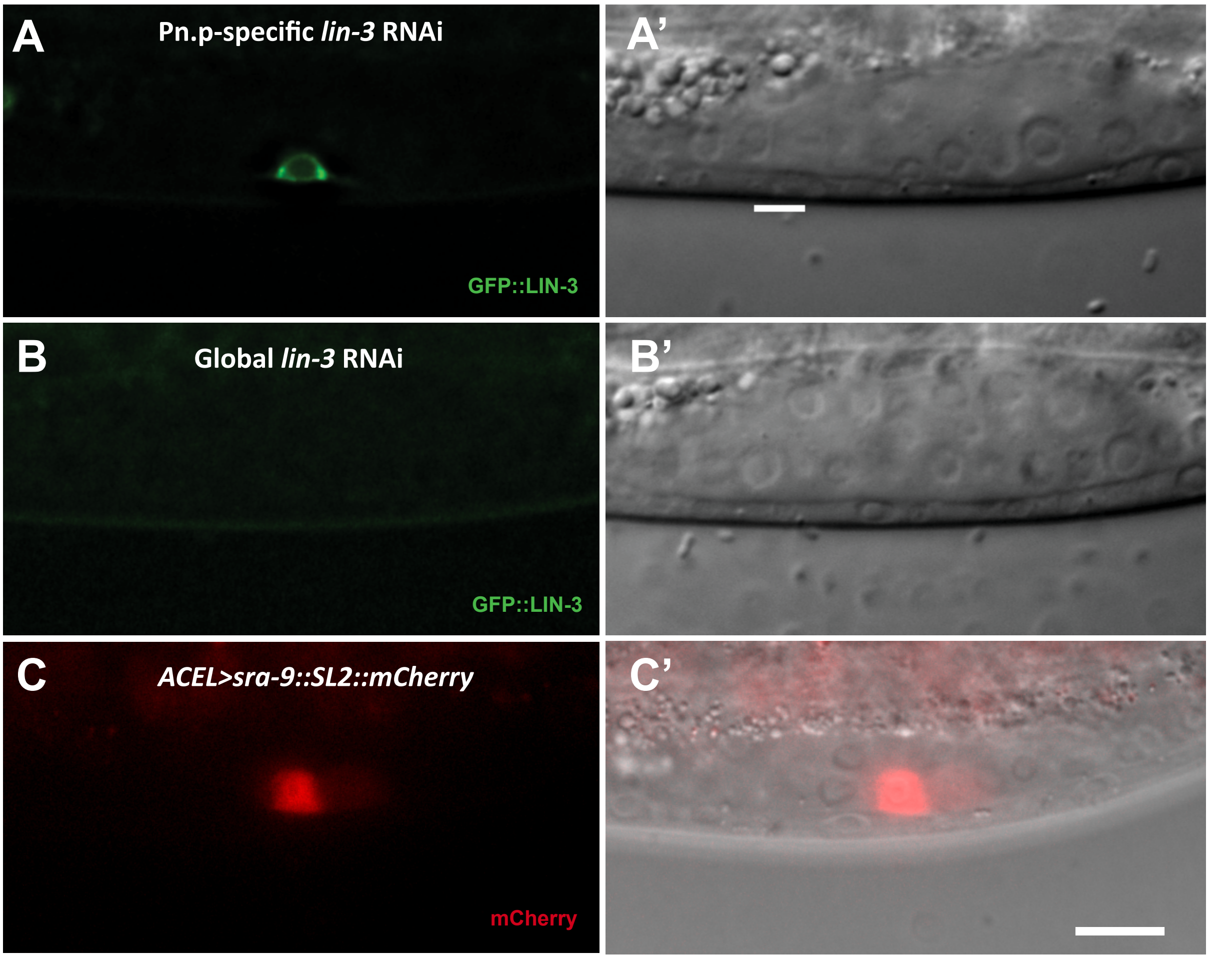

Figure S6. Specificity of the Pn.p-specific RNAi and the AC-specific rescue strains. (A,A') To monitor the specificity of the tissue-specific RNAi experiment, *lin-3* RNAi was performed in the tissue-specific RNAi strain *rrf-3(pk1426)II; unc-119(ed4)III; rde-1(ne219)V; zhEx418[lin-31::rde-1genomic; myo2-mcherry]; zhIs67[gfp::lin-3, unc-119(+)]*, which permits efficient gene knock-down in the Pn.p cells ((Yang et al., 2017) and (B,B') in the global RNAi strain *rrf-3(pk1426)II; unc-119(ed4)III; zhIs67[gfp::lin-3, unc-119(+)]*. Global *lin-3* RNAi caused the depletion of the GFP::LIN-3 signal in the AC and a Vul phenotype, while Pn.p cell-specific RNAi did not reduce GFP::LIN-3 expression in the AC and did not cause a Vul phenotype. (C,C') AC-specific expression of *sra-9::SL2::mCherry* from the single-copy MosSci transgene *zhIs143[P_{ACEL}>sra-9::SL2::mCherry]*. mCherry expression in a late L2 larva is shown. The scale bar in (C') is 10μm.

Development • Supplementary information

Supplementary methods

Plasmid constructs

pLM5 (*lin-3*(*zh112)* repair template)

The pDD268 plasmid (Dickinson et al., 2015) was digested with the AvrII and SpeI restriction enzymes, adding a linker to the 3' end of the fluorophore insertion site when recombined with the homology arms by means of the Gibson protocol (Gibson et al., 2009). The inserted homology arms were amplified by Phusion PCR from genomic *lin-3* DNA with the following oligonucleotide combinations: HA1: 0.7 kb with OLM181 (ACG TTG TAA AAC GAC GGC CAG TCG CCG GCA GAA GAG GTC ATA CAG CAA TGC ACA G) and OLM182 (CAT CGA TGC TCC TGA GGC TCC CGA TGC TCC GAG ACA CGA TTC TGA AAC TTT TAT TG) and 0.46 kb with HA2: OLM183(CGT GAT TAC AAG GAT GAC GAT GAC AAG AGA CCT TCG TGG TTT CGT CAA GAA CGT AG) and OLM184 (GGA AAC AGC TAT GAC CAT GTT ATC GAT TTC CAG ACC TAA TCA AAT GGC TAC CTT TGC).

pLM12 (SGN3A for *lin-3(zh112)*)

Single guide SG3A sequence gtt ctt gac gaa acc acg aa was cloned through NEB's Q5 Site-Directed Mutagenesis Kit into pDD162 (Addgene plasmid # 47549) with OLM177 (gtt ctt gac gaa acc acg aaG TTT TAG AGC TAG AAA TAG CAA G).

pLM13 (SGN3B for *lin-3(zh112)*)

Single guide SG3A sequence cag aat cgt gtc tcc ctt cg was cloned through NEB's Q5 Site-Directed Mutagenesis Kit into pDD162 with OSS1 (cag aat cgt gtc tcc ctt cgG TTT TAG AGC TAG AAA TAG CAA G).

Development • Supplementary information

Development • Supplementary information

pLM6 (*nlp-26(zh113)* repair template)

The pDD282 plasmid (Addgene #66823) was digested with the AvrII and SpeI restriction enzymes, adding a linker to the 3' end of the fluorophore insertion site when recombined with the homology arms by means of the Gibson protocol (Gibson et al., 2009). The inserted homology arms were amplified by Phusion PCR from genomic *nlp-26* DNA with the following oligonucleotide combinations: HA1: 0.6 kb with OSS3 (acg ttg taa aac gac ggc cag tcg ccg gca TTG GCG GGA AAT TCA ATG TTT CAG TC) and OSS4 (CAT CGA TGC TCC TGA GGC TCC CGA TGC TCC tAG GGA GAA GAT CAC GAA GAA GTT C) and 0.6 kb with HA2: OSS5 (CGT GAT TAC AAG GAT GAC GAT GAC AAG AGA CTT CTT GTT GGC CTT GTA TCC GCA C) and OSS6 (gga aac agc tat gac cat gtt atc gat ttc CTG CTT GGT GTT ATA TTT GAA GGG TAT CAC).

pLM8 (SGN2 for *nlp-26(zh113)*)

Single guide N2 ATA CAA GGC CAA CAA GAA GG was cloned through NEB's Q5 Site-Directed Mutagenesis Kit 2 with primers OSS7 (CCT TCT TGT TGG CCT TGT ATG TTT TAG AGC TAG AAA TAG CAA G) into plasmid pDD162 (Addgene # 47549).

pLM9 (SGN3 for *nlp-26(zh113)*)

Single guide N3 ATA CAA GGC CAA CAA GAA GG was cloned through NEB's Q5 Site-Directed Mutagenesis Kit with primers OSS8 (TAC AAG GCC AAC AAG AAG GAG TTT TAG AGC TAG AAA TAG CAA G) into plasmid pDD162.

pLM10 (SGN4 for *nlp-26(zh113)*)

Single guide N4 sequence ATA CAA GGC CAA CAA GAA GG was cloned through NEB's Q5 Site-Directed Mutagenesis Kit with primers OSS9 (TCT TGT TGG CCT TGT ATC CGG TTT TAG AGC TAG AAA TAG CAA G) into plasmid pDD162.

pSS22 (*sra-9(zh151)* repair template)

The pDD282 plasmid was digested with the AvrII and SpeI restriction enzymes, adding a linker to the 5' end of the fluorophore insertion site when recombined with the homology arms by means of the Gibson protocol (Gibson et al., 2009). The inserted homology arms were amplified by Phusion PCR from genomic *sra-9* DNA with the following oligonucleotide combinations: HA1: 0.666 kb with OSS181 (ACG TTG TAA AAC GAC GGC CAG TCG CCG GCA CTG TTG GAG TAG GGG CAT TGA GAC ATT TG) and OSS182 (CAT CGA TGC TCC TGA GGC TCC CGA TGC TCC ACT CCA CAT aTT tTT CAT tTG aCT gAT ATG GTT CTC CTG AG) and 0.686 kb with HA2: OSS179 (CGT GAT TAC AAG GAT GAC GAT GAC AAG AGA TAA CAA GTT TAA AAA AAT TTC ATT GGA ACT TGA AG) and OSS180 (CAG CTA TGA CCA TTT ATC GAT TTC GCA TCC GAA CGC AAT GAA CTT TTT GAG CTC AC)

pSS20 (SGN1 *sra-9(zh151)*)

Single guide N1 sequence GAT GAA GAA CAT GTG GAG TT by cycle restriction-ligation with the primers OSS175 (ctt gTT AGC CAG ATG AAG AAC ATG) and OSS176 (aaa cCA TGT TCT TCA TCT GGC TAA) into plasmid pDD162.

pSS21 (SGN2 *sra-9(zh151)*)

Single guide N2 sequence GAT GAA GAA CAT GTG GAG TT was cloned by cycle restriction-ligation with the primers OSS177 (ctt gGA TGA AGA ACA TGT GGA GTT)

Development • Supplementary information

and OSS178 (aaa cAA CTC CAC ATG TTC TTC ATC) into plasmid pDD162.

pMMO10 (*zhIs67[gfp::lin-3]*)

The *gfp* coding sequences amplified with the primers OJE131 (AGT CGA CCT GCA GGC ATG CAA GCT gag aca cga ttc tga aac) and OJE132 (GGC ATG GAT GAA CTA TAC AAA cct tcg tgg ttt cgt caa) were inserted into the *lin-3* locus amplified with primers OMMO118 (ttt cct agg CAT CGT TGA CTG ACT CAT G) and OMMO119 (ttt cct agg CGA CAT CAA GGT TCA CGG) by fusion PCR and subcloned into the AvrII site of pCFJ151 (Frøkjær-Jensen et al., 2008). The extrachromosomal array *zhEx523* was generated by injecting 50 ng/µl of plasmid MMO10 together with 10 ng/µl pGH8, 2.5 ng/µl pCFJ90, 5 ng/µl pCFJ104 and 50 ng/µl pJL43.1 as co-injection markers, and *zhEx523* was integrated by gamma-irradiation to generate the *zhIs67[gfp::lin-3]* reporter.

pMW87 (*zhIs143[*$P_{ACEL\ \Delta pes10}$*>sra-9*$_{genomic}$*::SL2::mCherry::unc-54 3'UTR])*

The anchor cell-specific enhancer element of *lin-3* (ACEL), which has been coupled to the minimal *Δpes-10* promoter, was amplified with GGT ACC AGA GCT CAC CTA GGC ACC TGT GTA TTT TAT GCT GG / GCA CAA GCT ATG GTA GCC ATA ATC AAT GCC TGA AAG TTA AAA ATT AC, and fused by Gibson assembly (Gibson et al., 2009) to the genomic region of *sra-9* (amplified with ATG GCT ACC ATA GCT TGT GCA TC / TTA ACT CCA CAT GTT CTT CAT CTG) and to *SL2::mCherry::unc-54 3'UTR* in pCFJ151 (amplified with GAA GAA CAT GTG GAG TTA AGC TGT CTC ATC CTA CTT TCA CCT AG / CCT AGG TGA GCT CTG GTA CCC TCT AG). Plasmid pMW87 was injected at a concentration of 50 ng/µl together with 10 ng/µl pGH8, 2.5 ng/µl pCFJ90, 5 ng/µl pCFJ104 and 50 ng/µl pJL43.1 as co-injection markers to generate a single-copy insertion on LGIII according to the MosSci protocol (Frøkjær-Jensen et al., 2008).

Development • Supplementary information

Development • Supplementary information

pMW89 (for *zhEx632[P*$_{nlp\text{-}26}$*::gfp::lacZ::unc-54 3'UTR]*)

1.43 kb from the *nlp-26* regulatory region was amplified with CTT GGA AAT GAA ATA AGC TTC CGT GTT TGT ATG AAT TGG CTG TG / CTT TGG CCA ATC CCG GGG ATC AAT TCT AGA AAT TTT TAG TAC AAA AAT TTC and cloned into the BamHI and HindIII sites of pPD96.04 by Gibson assembly (Gibson et al., 2009). Plasmid pMW89 was injected at a concentration of 100 ng/µl together with 50 ng/µl pBluescript KS- as carrier DNA and 2.5 ng/µl pCFJ90 as co-injection marker to generate the extrachromosomal array *zhEx632.*

pMW98 (for *zhEx668.1* to *zhEx668.3[P*$_{cdh\text{-}3}$*::unc-40*$_{minigene}$*::gfp::unc-54 3'UTR]*)

The minimal AC element *mk62-63* of the *cdh-3* regulatory region (Ziel et al., 2009) was amplified with CTT GGA AAT GAA ATC CTA GGT AGA GCA TGA TGT CCT TAC CTT G / CCG AAA TGT CGC AAA ATC ATA GCT CGG TAC CCT CCA AGC AAG and fused by Gibson assembly (Gibson et al., 2009) to the *unc-40*$_{minigene}$*::gfp::unc-54 3'UTR*, which had been amplified with GAT TTT GCG ACA TTT CGG TGA GTT C / GAC TCC AAG GAT GCG GAG TCT GTT CGG CTC AAT TAC AAA ATA C from genomic DNA and with ACT CCG CAT CCT TGG AGT CGT ACG / GTG CCA CCT GAC GTC TAA GAA ACC from *qyIs66* (Ziel et al., 2009), and cloned into the vector backbone of pPD95.75 (amplified with CTT AGA CGT CAG GTG GCA CTT TTC / ACG CTA ACA ACT TGG AAA TGA AAT). The mutation R938L that was present in the coding frame of the *unc-40* cDNA of *qyIs66* was corrected by site directed mutagenesis. Plasmid pMW98 was injected at a concentration of 50 ng/µl together with 150 ng/µl pBluescript KS+ as carrier DNA and 2.5 ng/µl pCFJ90 as co-injection marker to generate three independent extrachromosomal arrays *zhEx668.1* to *zhEx668.3.*
